# Curcumin Rewires the Tumor Metabolic Landscape: Mechanisms and Clinical Prospects

**DOI:** 10.3390/nu18010053

**Published:** 2025-12-23

**Authors:** Dingya Sun, Dun Hu, Jialu Wang, Xin Li, Jun Peng, Shan Wang

**Affiliations:** 1 Department of Pharmacology, Xiangya School of Pharmaceutical Sciences, Central South University, Changsha 410017, China; sundingya627@163.com; 2 Department of Pharmaceutical Engineering, College of Chemistry and Chemical Engineering, Central South University, Changsha 410017, China; huduncsu@163.com (D.H.); m16680808529@163.com (J.W.); 3 Hunan Provincial Key Laboratory of the Research and Development of Novel Pharmaceutical Preparations, College of Pharmacy, Changsha Medical University, Changsha 410017, China; tengyunxin2010@163.com

**Keywords:** curcumin, cancer metabolism, glucose and lipid metabolism, amino acid metabolism, mitochondrial energy metabolism

## Abstract

Metabolic reprogramming is a fundamental hallmark and a key driver of malignant tumors. By reshaping glucose, lipid, and amino acid metabolism, as well as mitochondrial function, it sustains the abnormal proliferation and survival of tumor cells, making it a crucial target for anti-tumor therapy. Curcumin, a natural multi-target compound, exhibits unique advantages in intervening in tumor metabolic reprogramming due to its low toxicity and broad-spectrum regulatory properties. In various tumor models, it can directly modulate the activity of key glycolytic enzymes, such as hexokinase 2, lactate dehydrogenase A, and pyruvate kinase M2, as well as transporters like glucose transporter 1. Furthermore, it inhibits the expression of proteins related to lipid metabolism, including fatty acid synthase and stearoyl-CoA desaturase 1, while also intervening in amino acid metabolic networks, such as glutaminase and branched-chain amino acid transaminase. Additionally, curcumin targets mitochondrial function and reactive oxygen species balance, creating multi-dimensional intervention effects through various pathways, including the induction of ferroptosis by regulating the SLC7A11/GPX4 axis and modulating gut microbiota metabolism. Its mechanism of action involves the synergistic regulation of key signaling pathways, including phosphoinositide 3-kinase/Akt, NF-κB, AMP-activated protein kinase, and hypoxia-inducible factor-1alpha. Furthermore, its specific effect profile demonstrates significant dependency on cell type and tumor model. This article systematically reviews the regulatory effects of curcumin on these critical metabolic processes and pathways in tumor metabolic reprogramming, revealing its molecular mechanisms in disrupting tumor growth and progression by targeting energy and biosynthetic metabolism. These findings provide a significant theoretical foundation and a preclinical research perspective for the development of natural antitumor drugs based on metabolic regulation, as well as for optimizing combination therapy strategies.

## 1. Introduction

Metabolic reprogramming of tumors represents a fundamental mechanism by which tumor cells dynamically adjust their metabolic networks to satisfy the demands of rapid proliferation and survival. This process ensures a steady supply of energy, supports biosynthesis, and facilitates adaptation to the microenvironment, establishing it as a new paradigm in cancer research and a critical therapeutic target [1]. Characteristic features include alterations in energy metabolism patterns, predominantly characterized by the ‘Warburg effect’ (i.e., aerobic glycolysis), alongside extensive remodeling of lipid metabolism, amino acid metabolism, and mitochondrial function. These metabolic alterations not only fulfill the biosynthetic precursor requirements of tumor cells but also significantly influence epigenetics, the immune microenvironment, and cell fate decisions via metabolite signaling [1].

This intricate process of metabolic remodeling is precisely and synergistically regulated by key signaling pathways, including PI3K/Akt/mechanistic target of rapamycin (mTOR), AMPK, mitogen-activated protein kinases (MAPK), and HIF-1α. For example, HIF-1α activates glycolytic enzymes and angiogenic factors under hypoxic conditions, while the mTOR pathway coordinates glucose transport and protein synthesis. Together, these pathways endow tumor cells with remarkable plasticity to adapt to metabolic stress [1]. Recent research has further revealed that gut microbiota and their metabolites, such as short-chain fatty acids, can influence tumor progression by modulating host energy metabolism and inflammatory responses [2,3]. Additionally, emerging concepts such as aberrant polyamine metabolism and ferroptosis have significantly broadened our understanding of the complexity of tumor metabolism [4,5].

Curcumin (diferuloylmethane) is a natural polyphenolic compound derived from the rhizomes of turmeric, widely recognized for its diverse pharmacological activities, including anti-inflammatory, antioxidant, and antimicrobial effects. The primary mechanism underlying its anticancer properties involves the multi-targeted regulation of key signaling pathways. This includes the inhibition of pro-cancer signals such as Akt/mTOR and NF-κB, as well as the activation of tumor-suppressive pathways like AMPK and p53, which collectively block cancer cell proliferation, invasion, and induce apoptosis [6]. Furthermore, curcumin also intervenes multidimensionally in metabolism by modulating gut microbiota, polyamine pathways, and related processes [2,4,5].

Although previous studies have systematically revealed the extensive regulation of curcumin on cancer cell signaling networks, its systemic impact on tumor metabolic reprogramming—an emerging hallmark of cancer—remains to be thoroughly elucidated. This article aims to focus on the dynamic intervention of curcumin in tumor metabolism by systematically reviewing the latest research progress on its remodeling of tumor energy and biosynthetic homeostasis. This remodeling occurs through the regulation of multiple aspects, including tumor cell glycolysis, lipid synthesis, amino acid metabolism, ferroptosis, mitochondrial function, and the microbiota-host metabolic axis. The findings provide new evidence for elucidating the multidimensional anticancer mechanisms of curcumin and lay a theoretical foundation for developing targeted therapeutic strategies based on tumor metabolic reprogramming. Tumor metabolic reprogramming is characterized by significant heterogeneity, influenced by various factors including tumor type, genetic background, and microenvironment. Consequently, the metabolic regulatory effects of curcumin discussed in this article must be understood within this complex context, as its specific mechanisms and intensity of effects may vary across different model systems. This review will focus particularly on these aspects.

## 2. Methods

This narrative review aims to systematically explore the molecular mechanisms by which curcumin inhibits tumor growth and progression through intervention in key metabolic pathways, including glucose metabolism, lipid metabolism, amino acid metabolism, and mitochondrial function.

### 2.1. Literature Search Strategy

The relevant literature was primarily retrieved from the PubMed database. The core search strategy involved pairing ‘curcumin’ with key terms related to tumor metabolism, including ‘tumor glucose metabolism,’ ‘tumor lipid metabolism,’ ‘tumor amino acid metabolism,’ ‘tumor mitochondrial metabolism,’ ‘ferroptosis,’ ‘gut microbiota,’ ‘Warburg effect,’ and ‘metabolic reprogramming.’ Additional specific keywords such as ‘glycolysis,’ ‘glutamine,’ ‘arachidonic acid metabolism,’ ‘polyamine metabolism,’ ‘reactive oxygen species,’ and ‘oxidative phosphorylation’ were also employed. The search encompassed publications from January 1990 to October 2025. To ensure comprehensive coverage, the reference lists of retrieved relevant reviews and key original articles were manually screened for additional eligible studies.

### 2.2. Study Selection (Inclusion and Exclusion Criteria)

This study established clear inclusion and exclusion criteria for literature screening. We primarily included preclinical studies (both in vitro and in vivo) and clinical investigations that examined any aspect of curcumin (or its derivatives/formulations) on tumor cell metabolism or metabolism-related cell death (such as ferroptosis). We incorporated original research articles, including experimental studies (cell culture, animal models) and clinical trials (any phase); relevant narrative reviews and systematic reviews were used solely for background understanding and reference tracking, not as primary evidence for mechanistic claims. Only peer-reviewed academic articles published in English were included, while conference abstracts, editorials, and non-research communications were excluded.

### 2.3. Data Extraction and Synthesis

A narrative synthesis approach was employed. Following the literature search, titles and abstracts were screened for relevance by one author, with uncertainties resolved through discussion with a second author. For included studies, key data were extracted into a structured template, including: study type (in vitro/in vivo/clinical), tumor model/cell line, curcumin formulation/dose, investigated metabolic pathway(s), main findings, and proposed mechanisms. Given the significant heterogeneity in experimental models (e.g., diverse cell lines, animal strains, curcumin formulations), study designs, and reported outcomes, a quantitative meta-analysis was deemed inappropriate. Instead, the extracted evidence was thematically categorized (e.g., effects on glucose metabolism, lipid metabolism, ferroptosis) for comparative analysis and narrative summary.

### 2.4. Consideration of Evidence Quality and Potential Biases

As a narrative review, this study did not conduct a formal systematic quality assessment or evidence grading (e.g., using GRADE tools) for the included individual studies. However, we acknowledge several potential sources of bias within the evidence base and the synthesis process itself. First, most evidence was derived from preclinical in vitro studies, which may not fully replicate the complexity of the human tumor microenvironment, thus introducing study design bias. Second, publication bias may have overestimated the true effects of curcumin, as positive results demonstrating significant effects are more likely to be published than null or negative findings. Additionally, our reliance on English-language literature may have led to selection bias, potentially omitting relevant studies published in other languages. Within individual studies, selective outcome reporting bias may also exist. In the synthesis process, we strive to present a balanced perspective by incorporating studies that report positive, negative, and null results on specific pathways, when such data are available. These limitations have been considered in the interpretation of results and will be explicitly discussed in the “Limitations” section of the conclusion chapter.

## 3. The Anti-Tumor Effects of Curcumin

**Figure 1:**
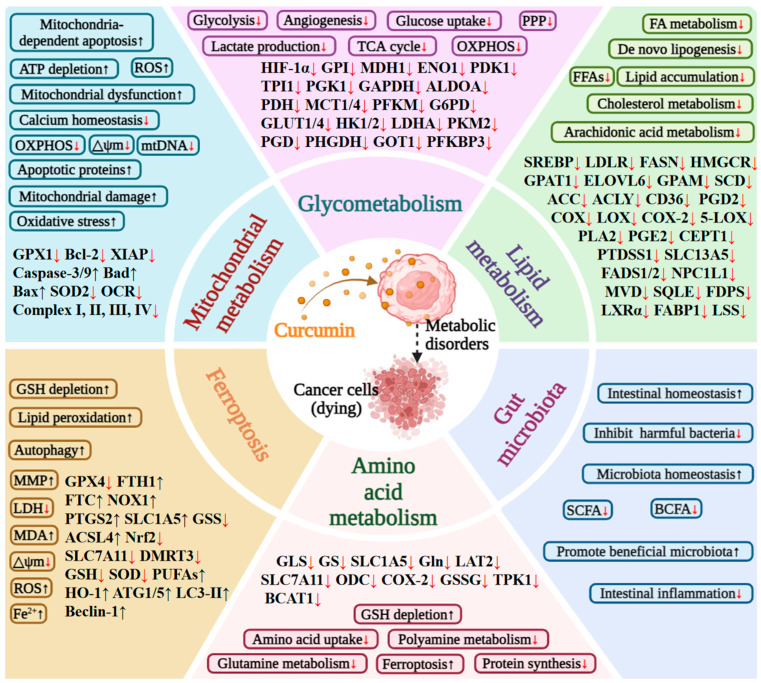
Curcumin exerts its antitumor effects by regulating tumor metabolism through multiple targets. Curcumin synergistically inhibits key signaling pathways, including PI3K/Akt and AMPK, while also targeting glucose metabolism by inhibiting the Warburg effect. It blocks lipid synthesis, interferes with amino acid utilization, disrupts mitochondrial function, induces ferroptosis, and reshapes the metabolism of gut microbiota. This multifaceted approach ultimately disrupts the energy and biosynthetic supply of tumor cells, effectively inhibiting tumor growth. Colored arrows indicate the inhibitory (red) or stimulatory (black) effects of curcumin on downstream signaling targets, finally involved in regulating a variety of intracellular physiological processes (see text for more details). (Original drawing, created with BioRender, https://biorender.com).

Curcumin, a multi-target natural compound, exerts broad-spectrum anti-tumor effects by synergistically regulating multiple key signaling pathways in tumor cells [6] (Figure 1). Its core mechanism involves the bidirectional regulation of cellular signaling networks: on one hand, it inhibits pro-tumorigenic signals such as Akt/mTOR, NF-κB, Wnt/β-catenin, and STAT3, thereby blocking tumor proliferation, invasion, and metastasis; on the other hand, it activates tumor-suppressive and cytoprotective pathways, including AMPK, p53, and nuclear factor erythroid 2-related factor 2 (Nrf2), which induce cell cycle arrest and apoptosis [7,8]. Consequently, curcumin can directly trigger various cell death programs, including apoptosis through modulation of the Bcl-2/Bax ratio and activation of the Caspase cascade [6], as well as ferroptosis by inhibiting the solute carrier family 7 member 11 (SLC7A11)/glutathione peroxidase 4 (GPX4) axis [5]. Moreover, it reshapes cellular homeostasis by activating autophagy and inhibiting epigenetic regulatory mechanisms such as DNMTs and HDACs. The anti-tumor effects of curcumin also extend to the tumor microenvironment, where it antagonizes angiogenesis by inhibiting VEGF signaling and enhances the immune microenvironment by improving T cell and NK cell function [9,10,11].

The multi-dimensional intervention of curcumin in tumor metabolic reprogramming serves as a crucial mechanism for disrupting the energy supply and biosynthetic homeostasis of tumors (Figure 1). Curcumin effectively reverses the Warburg effect and interferes with glucose metabolism by targeting HIF-1α and inhibiting key enzymes, including hexokinase 2 (HK2), pyruvate kinase M2 (PKM2), and lactate dehydrogenase A (LDHA), as well as the functions of glucose transporter 1 (GLUT1) and monocarboxylate transporters (MCTs) [12]. Additionally, in the context of lipid metabolism, curcumin activates signaling pathways such as AMPK, downregulates fatty acid synthase (FASN) and stearoyl-CoA desaturase (SCD1), and affects key proteins involved in cholesterol metabolism, thereby inhibiting de novo lipogenesis (DNL) [13]. Curcumin extensively intervenes in amino acid metabolism by inhibiting the activity of glutaminase (GLS), ornithine decarboxylase (ODC), and other enzymes, thereby disrupting polyamine homeostasis and amino acid balance, which affects tumor proliferation and antioxidant defense [14]. Furthermore, curcumin can induce mitochondrial dysfunction, impacting energy metabolism and inducing apoptosis through mechanisms such as disrupting the electron transport chain, reducing membrane potential, and promoting the generation of reactive oxygen species (ROS). Additionally, by regulating key molecular pathways such as acyl-CoA synthetase long-chain family member 4 (ACSL4) and SLC7A11/GPX4, curcumin induces lipid peroxidation and collapses redox homeostasis, thereby activating the ferroptosis program [15]. Moreover, curcumin can indirectly inhibit tumor progression by reshaping gut microbiota metabolism, enriching probiotics, suppressing pathogenic bacteria, and modulating the microbiota-host metabolic axis, including short-chain fatty acids and bile acid metabolism [16].

The current study further elucidates that curcumin’s intervention in tumor metabolic reprogramming serves as a crucial mechanism for disrupting the energy supply and biosynthetic homeostasis of tumors, thereby reversing chemotherapy resistance (Figure 1). This finding provides a more profound mechanistic explanation for its multi-target anticancer effects and underscores its significant potential and clinical applicability as a natural metabolic regulator.

## 4. Curcumin Regulates Tumor Glucose Metabolism

### 4.1. Tumor Glucose Metabolism and the Warburg Effect

As the primary energy and material source for cancer cells, glucose is metabolized via the Warburg effect, also known as aerobic glycolysis. The intermediates produced in this metabolic pathway not only serve as precursors for the synthesis of macromolecules, such as lipids, amino acids, and nucleic acids, but also fulfill the energy requirements of rapid cell proliferation through ATP generation [1]. The end product of glycolysis, lactate, plays a dual role by remodeling the tumor microenvironment and synergistically inducing an immunosuppressive state. At the molecular level, oncogenes drive tumor progression by activating the glycolysis-lactate metabolic axis, which is facilitated by the regulation of key metabolic enzymes, ultimately promoting tumorigenesis and development [1]. Extensive research has shown that targeted inhibition of this metabolic axis can effectively reverse the Warburg effect phenotype in tumors.

### 4.2. Targeting HIF-1α: A Core Mechanism of Curcumin in Regulating Metabolic Reprogramming

Hypoxia-induced abnormal accumulation of HIF-1α within the tumor microenvironment drives malignant progression through the regulation of metabolic reprogramming. As a central metabolic hub, HIF-1α not only upregulates the expression of glycolytic enzymes and transporters to promote the Warburg effect but also mediates angiogenesis and therapy resistance, with its overexpression significantly correlating with poor clinical prognosis. Curcumin intervenes in this process by inhibiting both the transcriptional activity and protein stability of HIF-1α [9,17,18] (Figure 1, Table 1). In hepatocellular carcinoma (HCC) cells, curcumin inhibits tumor invasion and metastasis by disrupting the HIF-1α-mediated epithelial–mesenchymal transition (EMT) process [18]. In human pituitary adenomas, curcumin suppresses hypoxia-induced mRNA and protein synthesis of HIF-1α and VEGFA, significantly reducing angiogenesis [9]. In cervical cancer (CC) models, tetrahydrocurcumin—one of the major metabolites of curcumin in vivo—exhibits potent anti-angiogenic effects by targeting the HIF-1α/VEGF-VEGFR-2 axis [19]. Furthermore, curcumin exerts multidimensional therapeutic effects by targeting the hypoxia-HIF-1α signaling pathway, thereby inhibiting tumor metabolic adaptation, blocking invasion and metastasis, and enhancing chemosensitivity. In hypoxic pancreatic cancer cells, curcumin downregulates the expression of GLUT1, HK2, LDHA, and PDK1 by inhibiting the Beclin1/HIF-1α axis, which results in reduced ATP production and inhibited cell proliferation [20]. Notably, PDK1 impedes the conversion of pyruvate to acetyl-CoA through the phosphorylation of pyruvate dehydrogenase (PDH), thereby obstructing the tricarboxylic acid cycle (TCA) and sustaining aerobic glycolysis. In TRAIL-resistant clear cell renal cell carcinoma (ccRCC) cells, curcumin also inhibits aerobic glycolysis and reverses therapeutic resistance by upregulating miRNA Let-7C, which targets the HIF-1α and PDK1 signaling pathways [21].

### 4.3. Direct Inhibitory Effects on Key Metabolic Enzymes and Transporters

In various studied tumor cell lines, curcumin has demonstrated potent antitumor effects by directly targeting key enzymes involved in glucose metabolism and inhibiting the Warburg effect (Figure 1, Table 1). Research on the targeting of HK2 is relatively comprehensive. In colorectal cancer (CRC) and acid-resistant prostate cancer cells, curcumin directly downregulates HK2, thereby inhibiting aerobic glycolysis [23,25]. In liver cancer models, curcumin reduces HK2 expression by inhibiting the activity of CSN5 kinase, which decreases glycolytic flux and invasive capacity, effectively prolonging the survival of tumor-bearing animals [27]. In breast cancer (BC) cells, curcumin enhances sensitivity to 4-hydroxytamoxifen and induces apoptosis by inhibiting the SLUG/HK2 axis [31].

The regulation of PKM2 by curcumin represents a fundamental mechanism in cancer biology. The elevated expression of PKM2, which is characteristic of cancer cells, serves as a primary driver of the Warburg effect. Curcumin alters the PKM splicing pattern by inhibiting DNA methyltransferase (DNMT3B), thereby promoting the conversion of PKM2 to the predominant PKM1 isoform, which facilitates oxidative metabolism in normal cells. This process is particularly evident in head and neck cancer cells, where curcumin effectively reduces glucose uptake and lactate production, leading to tumor growth inhibition and apoptosis [37,48]. Furthermore, in various cancer types, including lung, breast, cervical, and prostate cancers, curcumin downregulates PKM2 through the inhibition of the mTOR/HIF-1α signaling pathway, which disrupts glucose uptake and lactate release. Concurrently, the expression of glucose transporter GLUT1 and HK2 is significantly reduced, resulting in a marked attenuation of the Warburg effect [17,49].

Curcumin exhibits broad-spectrum effects by directly binding to and inhibiting multiple metabolic enzymes. Quantitative proteomics reveals that curcumin binds to multiple glycolytic and pentose phosphate pathway enzymes (e.g., GAPDH, PKM1/2, LDHA, MDH1/2, PGD, PHGDH, TPI, PGK1, ALDOA), thereby disrupting metabolic flux [12]. Specifically, in glioma cells, curcumin inhibits G6PT activity, leading to energy depletion and subsequent cell death [34]. In prostate cancer, it reduces both the activity and expression of PDH, consequently inhibiting the TCA cycle and acetyl-CoA production [26]. Additionally, in melanoma cells, curcumin significantly decreases G6PD activity, thereby regulating the pentose phosphate pathway and suppressing tumor growth [39]. Furthermore, the curcumin analog MS13 also demonstrates therapeutic effects in gliomas and neuroblastomas by inhibiting the expression of ENO1, GAPDH, TPI1, and PGK1 [36].

Curcumin exhibits significant efficacy in inhibiting metabolic transporters. Its treatment leads to a marked downregulation of GLUT1, MCT1, and MCT4 expression in HCC cells, concurrently inhibiting cellular glucose consumption, lactate efflux, and extracellular acidification [28,29,30]. In BC models, curcumin suppresses GLUT1 expression via the PPARδ/Akt signaling pathway [32,33]. Liao et al. reported that curcumin can also significantly inhibit the invasion and metastasis of lung cancer cells by targeting the GLUT1/MT1-MMP/MMP2 signaling pathway [47]. In melanoma, the analog GO-Y030 downregulates glucose transporters (GLUT1, GLUT4), PDK1, and PFKM, leading to a significant reduction in glucose uptake and glycolysis [38].

### 4.4. Coordinated Inhibition of Metabolic Reprogramming Through Signaling Pathway Networks

In addition to its direct targeting capabilities, curcumin significantly modulates tumor metabolic reprogramming by synergistically inhibiting multiple upstream signaling pathway networks. It exerts its effects by targeting key oncogenic signaling axes (Figure 1, Table 1). In prostate cancer cells, curcumin inhibits the activation of the ERK1/2/c-Myc/HIF-1α signaling axis and reduces the activity of rate-limiting enzymes such as HK1/2, PFKP, and PDH in a concentration-dependent manner, thereby blocking glycolytic metabolic flux and glucose consumption [26]. In chronic myeloid leukemia cells, curcumin inhibits HIF-α activity through the miR-22/IPO7/HIF-1α signaling axis, which synergistically downregulates the enzymatic activities of multiple glycolytic enzymes, pentose phosphate pathway enzymes, and HIF-1α target genes [40]. In lymphoma models, curcumin downregulates LDHA activity and expression by targeting the c-Myc and HIF-1α signaling pathways, thereby reducing glycolysis and inhibiting tumor progression [41].

Curcumin also targets energy-sensing and non-classical pathways. For example, in esophageal squamous cell carcinoma, it downregulates GLUT4, HK2, PFKBP3, and PKM2 expression in an AMPK-dependent manner [42]. In papillary thyroid carcinoma (PTC) cells, curcumin modulates Akt to decrease the expression of LINC00691, subsequently inhibiting the activity of LDHA and HK2, which reduces glucose uptake and lactate production, ultimately leading to apoptosis [43].

The induction of ROS generation constitutes another critical mechanism by which curcumin exerts its effects. In gastric cancer (GC) cells, curcumin induces mitochondrial dysfunction by facilitating a significant increase in ROS levels, which markedly diminishes both oxidative phosphorylation (OXPHOS) and glycolytic activities [45]. Furthermore, its analog Z35 demonstrates strong inhibitory effects on glycolysis, mediated through the activation of the ROS/YAP/JNK pathway [44].

Moreover, curcumin has been shown to effectively reverse the metabolic adaptability of tumors. In the context of the high-glucose microenvironment characteristic of tumors, curcumin counteracts EGF-induced activation of the EGFR/ERK/Akt signaling pathway in pancreatic cancer cells, thereby inhibiting cell proliferation and invasion [22]. In HCC cells, curcumin mitigates the increase in glucose consumption, lactate efflux, and the extracellular acidification phenotype, while also reducing the production of nitric oxide (NO) and ROS. These effects are associated with its inhibitory action on the expression of several metabolic enzymes and transporters (such as GLUT1, MCT1, MCT4, HCAR-1, and MDR1) [30]. More importantly, curcumin can also reverse the activation of high glucose-induced pro-metabolic transcription factors and signaling molecules, including HIF-1α, mTOR, MYC, and STAT3, demonstrating a robust ability to target and disrupt the energy metabolic adaptability of tumors [30].

### 4.5. Metabolic Compensation Mechanisms and Treatment Resistance

The tumor metabolic compensation mechanism is a significant contributor to drug resistance, and curcumin intervention can activate this mechanism. For example, in adrenocortical carcinoma cells, curcumin partially upregulates glycolysis-related genes such as HK1, HK2, and PFKL by downregulating ERRα expression. However, the conversion of the glycolysis end product, pyruvate, to lactate, as well as the flux through the TCA cycle, is markedly restricted. This is evidenced by the simultaneous inhibition of LDHA activity, the extracellular acidification rate (ECAR), and the oxygen consumption rate (OCR), resulting in a dual reduction in both glycolytic flux and metabolic reserve capacity [46]. This phenomenon indicates that cancer cells are attempting to counteract the therapeutic pressure imposed by curcumin by selectively activating glycolysis and glutamine metabolism [46].

Similar metabolic reprogramming is observed in other models. Proteomic analysis of BC cells subjected to combined treatment, following enhanced curcumin uptake via electroporation (EP), revealed a significant reduction in the activity of glycolytic enzymes as well as in the pentose phosphate pathway. Conversely, there was an upregulation in the expression of enzymes associated with the TCA cycle and the OXPHOS pathway [35]. This metabolic reprogramming, characterized by a shift from glycolysis to the TCA cycle and OXPHOS, indicates that tumor cells, when subjected to glycolytic inhibition and a metabolic stress response, may sustain energy homeostasis by activating alternative pathways, such as glutamine metabolism [35]. This metabolic plasticity is a critical feature that enables cancer cells to evade therapy, underscoring the necessity of targeting multiple metabolic pathways in future treatments.

The antitumor metabolic regulatory effects of curcumin appear to exhibit cell-type specificity, closely associated with the intrinsic metabolic background of tumor cells and experimental conditions. Notably, the solubility and treatment conditions of curcumin, particularly the choice of solvent, are critical factors influencing these effects. For instance, in squamous cell carcinoma lines such as FaDu, Detroit 562, and Cal27, as well as colon cancer HT-29 cells, ethanol-dissolved curcumin consistently reduces glucose uptake. However, it exerts a bidirectional regulation on lactate production: lactate production increases in HT-29 cells, while both glucose uptake and lactate production increase synchronously in Detroit 562 and Cal27 cells. The variations in pharmacological efficacy are related to the baseline levels of the Warburg effect inherent in these cells. Specifically, Cal27 cells, which exhibit the lowest sensitivity, also display the lowest basal metabolic level, whereas HT-29 cells, which are the most resistant, show the highest basal metabolic level. It is noteworthy that the choice of solvent can significantly influence metabolic phenotypes [24]. For example, curcumin dissolved in DMSO consistently inhibits glycolysis in lung and CC cells via the mTOR/HIF-1α-PKM2 axis [17]. In contrast, ethanol as a solvent may regulate the activity of metabolic enzymes at the transcriptional level in tumor cells, producing effects that are opposite to those of curcumin and leading to contradictory metabolic phenotypic outcomes [24]. This indicates that analysis solely at the molecular level may misrepresent the direction of metabolic flux. Therefore, establishing a standardized experimental system (uniform solvent/concentration) and designing precise intervention strategies in conjunction with tumor metabolic heterogeneity is essential to enhance clinical applications. Future in vitro mechanistic studies must rigorously incorporate solvent control groups and provide comprehensive details regarding the type and final concentration of solvents used. Ideally, researchers should explore and develop more physiologically relevant delivery methods to minimize solvent interference, thereby enabling a more accurate assessment of the inherent metabolic regulatory effects of curcumin.

In summary, curcumin effectively disrupts the energy metabolic homeostasis of tumor cells by targeting key glycolytic enzymes, metabolic transporters, and signal transduction networks. Additionally, it weakens the metabolic adaptability of cancer cells and reverses treatment resistance. This provides a significant theoretical foundation and translational medical value for developing anti-tumor strategies based on metabolic regulation. Notably, curcumin continues to exert antioxidant effects and downregulate stress gene expression even after treatment discontinuation. Furthermore, it inhibits glycolytic metabolism, thereby reducing the formation of new blood vessels in the livers of tumor-bearing mice. However, whether its long-term anti-tumor effects are due to metabolite accumulation or the intrinsic action of the drug itself requires further investigation [50].

Although the phenomenon of metabolic compensation has been reported in multiple studies, indicating the metabolic plasticity of tumor cells under curcumin pressure, its specific manifestations—such as enhanced glycolysis, a shift to glutamine metabolism, or activation of oxidative phosphorylation (OXPHOS)—demonstrate significant context dependency. This dependency may arise from variations in the inherent metabolic foundations, genetic backgrounds, and microenvironmental factors of tumor cells. Therefore, it is crucial to consider metabolic compensation as a dynamic and conditional adaptive response rather than a singular, fixed outcome. This perspective is essential for understanding the therapeutic effects of curcumin and for designing subsequent combination strategies.

### 4.6. Considerations of Cell Type Dependency and Heterogeneity

It is important to emphasize that the regulatory effects of curcumin on glucose metabolism discussed in this chapter are primarily derived from studies conducted on specific cell lines and animal models. The inherent metabolic state of tumor cells, such as the baseline level of the Warburg effect, genetic variations including p53 status and PI3K mutations, and the microenvironment, particularly the extent of hypoxia, collectively influence their response to curcumin. For example, the solvent-dependent effects and the contradictory results regarding lactate production observed across different cell lines, as mentioned in Section 4.5, underscore this heterogeneity. Consequently, it is inappropriate to directly extrapolate the metabolic effects observed in any single study to all tumor types. Future research should prioritize validation in broader preclinical models that encompass well-defined molecular subtypes and the exploration of biomarkers that determine therapeutic sensitivity.

## 5. Curcumin Regulates Tumor Lipid Metabolism

Curcumin exhibits significant antitumor effects across various tumor models by modulating lipid metabolism pathways through multiple targets (Figure 1, Table 2). This chapter will review the most recent research advancements regarding curcumin’s role in regulating fatty acid synthesis, cholesterol metabolism, its synergistic effects with chemotherapeutic agents, and its potential in ameliorating cancer cachexia.

### 5.1. Inhibitory Effects on Fatty Acid Synthesis Metabolism

Curcumin effectively inhibits DNL in various tumor cells through key signaling pathways and transcription factors (Figure 1, Table 2). In HCC cells, curcumin activates AMPK and PPARα, while downregulating the lipogenic factor SREBP-1c and its target gene FAS. This action suppresses lipid synthesis and promotes lipolysis in HepG2 cells, leading to a reduction in triglyceride (TG) and total cholesterol (TC) accumulation [51]. The inhibition of FASN by curcumin is consistent across different cancer types, as it significantly reduces FAS activity and its mRNA and protein expression in both BC and HCC cells [59]. Additionally, in prostate cancer cells, curcumin modulates fatty acid and cholesterol metabolic pathways by downregulating the expression of lipid metabolism-related genes, including SREBP-1/2, LDLR, M-CIC, HMGCR, and GPAT [13]. Spatial metabolomics further confirms curcumin’s broad inhibitory effect on fatty acid synthesis enzymes, such as FASN, SCD, ELOVL1, GPAM, CEPT1, and PTDSS1 [55,60]. Its regulatory effects extend to the composition of cell membrane lipids, with research indicating that curcumin can reconstitute the membrane fatty acid profile in BC cells, potentially influencing membrane fluidity and signal transduction functions [69]. Notably, the curcumin analog demethoxycurcumin (DMC) significantly reduces lipid synthesis and total intracellular fatty acid content in prostate cancer and triple-negative BC cells by upregulating AMPK activity, which decreases FASN expression and inhibits ACC activity [58,62]. In HCC cells, curcumin downregulates the expression of SLC13A5 and ACLY via the AMPK-mTOR signaling axis, thereby synergistically reducing DNL and lipid accumulation [52]. Furthermore, the activation of AMPK signaling by curcumin not only inhibits lipid synthesis but also initiates a broader metabolic reprogramming. For instance, in lung adenocarcinoma cells, curcumin induces autophagy through the activation of AMPK signaling and subsequent phosphorylation of its downstream target, acetyl-CoA carboxylase (ACC), thereby expanding the mechanistic understanding of its tumor growth suppression via metabolic regulation [64]. Additionally, curcumin regulates the expression of enzymes involved in the biosynthesis of unsaturated fatty acids in mammary stem cells, including stearoyl-CoA desaturase (SCD), fatty acid desaturase 2 (FADS2), and fatty acid desaturase 1 (FADS1), which may serve as potential biomarkers—especially SCD—for assessing the efficacy of curcumin in BC prevention and treatment [70]. Simultaneously, curcumin significantly reduces SCD expression in myeloma cells [63]. From the perspective of lipid synthesis pathways, FASN serves as the key enzyme that catalyzes both the initial and the sole terminal steps of de novo lipid synthesis. This suggests that its inhibition may exert a ‘bottleneck’ effect on lipid flux. In contrast, SCD1 catalyzes the production of monounsaturated fatty acids, and its inhibition may more directly influence membrane fluidity and signal transduction. Curcumin simultaneously targets these nodes, potentially generating synergistic inhibitory effects. However, there is currently a lack of studies directly comparing the differences in antitumor efficacy between inhibiting individual targets and curcumin’s multi-target intervention.

### 5.2. Regulatory Effects on Cholesterol Metabolism

Curcumin exhibits significant regulatory potential on the absorption, synthesis, and excretion of cholesterol metabolism in tumor cells. In human HCC cells, curcumin treatment leads to a marked downregulation of the mRNA expression of SREBP and its target genes. This includes nine genes related to fatty acid and triglyceride synthesis (such as SREBP-1, FAS, ACC1, and SCD-1) and eleven genes associated with cholesterol synthesis (such as SREBP2, HMGCR, and HMGCS). These findings demonstrate curcumin’s strong potential to inhibit fatty acid and cholesterol synthesis metabolism [56]. In colon and liver cancer cells, curcumin reduces cholesterol absorption in a dose-dependent manner by inhibiting the expression of NPC1L1, SREBP-2, and HNF1α [53,65]. This mechanism also involves the stimulation of Ca^2+^ influx through the activation of the TRPA1 channel, which further reduces cholesterol absorption via the PPARγ/SP-1/SREBP-2/NPC1L1 signaling cascade, thereby synergistically inhibiting the proliferation of Caco-2 cells [66]. Notably, The study conducted by Peschel et al. reported a significant concentration-dependent increase in LDL receptor (LDLR) mRNA expression in hepatocellular carcinoma (HCC) cells following curcumin treatment, which may play a crucial role in its cholesterol-lowering effects. Notably, a moderate increase in the expression of HMG-CoA reductase, FDPS, and SREBP2 was observed only at higher concentrations of curcumin. Conversely, the expression of LXRα and its target gene ABCG1 exhibited an increase even at lower concentrations. Furthermore, curcumin was found to downregulate the PPARα target genes CD36 and FABP1. Collectively, these alterations in gene expression align with the cholesterol-lowering properties attributed to curcumin [54]. Animal experiments further corroborated the cholesterol-lowering effect of curcumin. In a rat model of DMH-induced colon cancer, treatment with curcumin and its analog BDMC-A significantly reduced cholesterol levels in the colon and intestines, enhanced the excretion of cholesterol and bile acids in feces, and reversed intestinal lipid accumulation. This effect was linked to the restoration of phospholipid content in the intestines and the inhibition of phospholipase A (PLA) and phospholipase C (PLC) activities [68].

### 5.3. Chemosensitization and Amelioration of Cancer Cachexia

Curcumin not only enhances the efficacy of chemotherapy by modulating tumor metabolism but also exhibits considerable potential in alleviating systemic metabolic disorders induced by tumors, particularly cancer cachexia. Regarding chemosensitization, studies indicate that curcumin can effectively amplify the antitumor effects of conventional chemotherapeutic agents and counteract drug resistance. In a mouse model of HCC, treatment with curcumin significantly decreased the levels of metabolic markers, including lactate dehydrogenase (LDH), triglycerides (TG), and FASN in serum, while upregulating the mRNA expression of high-density lipoprotein cholesterol (HDL-C) and apolipoprotein A1 (ApoA1). By inhibiting aerobic glycolysis (e.g., by suppressing LDH/HIF-1α activity) and lipid anabolism (e.g., by reducing levels of hexadecanoic acid and FASN), curcumin markedly enhances the anti-tumor efficacy of sorafenib [57]. Furthermore, curcumin can potentiate the anti-cancer effects of statins. In breast, colon, and liver cancer cells, it significantly suppresses the expression of cholesterol biosynthesis genes such as ELOV6, CYP51, LSS, MVD, SQLE, and FASN [61]. This finding provides a preclinical theoretical basis for the exploration of combination therapy strategies.

Curcumin presents a unique therapeutic strategy for addressing the challenges of fat and muscle atrophy associated with cancer cachexia. Research has demonstrated that in a mouse model of colon cancer cachexia, curcumin inhibits the activation of the cAMP/PKA/CREB signaling pathway in epididymal white adipose tissue (eWAT). It also downregulates the expression of key lipolysis proteins, including hormone-sensitive lipase (HSL), adipose triglyceride lipase (ATGL), and uncoupling protein 1 (UCP1), while reversing the cachexia-induced downregulation of FASN. These effects lead to a significant reduction in serum levels of free fatty acids (FFA), an increase in triglyceride reserves, and a marked amelioration of cachexia-induced weight loss and fat atrophy symptoms [67]. The dual regulatory effect of curcumin on body metabolism—by inhibiting catabolism and promoting anabolism—underscores its considerable potential as an adjunctive therapeutic agent in cancer treatment.

### 5.4. Curcumin Regulates Tumor Arachidonic Acid Metabolism

Abnormal arachidonic acid metabolism represents a critical pathological mechanism in inflammatory responses and tumorigenesis. Following the catalytic hydrolysis of membrane phospholipids by phospholipase A2 (PLA2), arachidonic acid is generated within cells and subsequently undergoes a series of metabolic processes through three primary enzymatic pathways: the cyclooxygenase (COX) pathway, the lipoxygenase (LOX) pathway, and the cytochrome P450 metabolic pathway [71]. Research has shown that selectively inhibiting rate-limiting enzymes, such as COX-2, can effectively obstruct abnormal metabolic pathways, thereby providing significant intervention targets for cancer prevention and treatment. Additionally, curcumin can modulate abnormal arachidonic acid metabolism in tumor cells by inhibiting the aforementioned enzyme systems, thereby exerting substantial anti-tumor pharmacological activity (Figure 1, Table 3).

#### 5.4.1. Direct Inhibition of Arachidonic Acid Metabolic Enzymes and Products by Curcumin

Curcumin exerts broad-spectrum antitumor effects by directly regulating arachidonic acid metabolism, which is manifested through the synergistic inhibition of related metabolic enzymes, including COX, LOX, and cPLA2, as well as downstream inflammatory mediators (Figure 1, Table 3). At the cellular level, multiple studies have confirmed curcumin’s direct inhibitory effects. In colon cancer cells, curcumin effectively blocks the release of arachidonic acid metabolites by inhibiting the phosphorylation of phospholipase A2 (cPLA2) and the activities of cyclooxygenase-2 (COX-2) and 5-lipoxygenase (5-LOX) [72]. This effect has been validated across various cell models, including human colon cancer HCT-116 cells, esophageal squamous cell carcinoma cells (KYSE-150 and KYSE-450), and immortalized intestinal epithelial IEC-6 cells. Notably, curcumin significantly inhibits the A23187-induced release of arachidonic acid in all these cell models [72]. Curcumin has been shown to activate the mitochondrial apoptosis pathway in acute myeloid leukemia cells by downregulating COX-2 expression [76]. In BC and its drug-resistant variants, curcumin inhibits tumor proliferation through the reduction in Bcl-2 and COX-2 expression [77]. Furthermore, in lung cancer cells, curcumin directly inhibits the activity of microsomal PGE2 synthase-1, thereby blocking the conversion of PGH2 to PGE2 [79]. In lung adenocarcinoma and pancreatic cancer cell lines, curcumin reduces the activity and expression of COX-2, EGFR, and ERK1/2 in a dose-dependent manner, which results in the inhibition of cell survival and the promotion of apoptosis [81]. Additionally, in CC cells, curcumin exerts anti-tumor effects by downregulating the expression of NF-κB, COX-2, and AP-1 [84].

Animal models and clinical studies further substantiate the direct inhibitory effects of curcumin. In a rat model of colon cancer, a diet supplemented with curcumin significantly reduced the production of prostaglandins (including PGE2, PGE2α, PGD2, and 6-keto-PGF1α) and thromboxane (TxB2) in liver and colon tissues. Additionally, it inhibited the formation of LOX pathway products such as 5(S)-, 8(S)-, 12(S)-, and 15(S)-HETEs, thereby markedly decreasing precancerous lesions in the colon [74]. Further investigations have demonstrated that curcumin inhibits both the COX and LOX metabolic pathways by downregulating the levels of phospholipase A2, phospholipase Cγ1, and PGE2 in colonic mucosa and tumor tissues, significantly suppressing tumor invasion and disease progression [75]. In various tumor animal models, curcumin has exhibited significant anti-angiogenic and anti-tumor effects. In liver cancer models, curcumin inhibits tumor angiogenesis by downregulating COX-2 and VEGF signaling pathways [90]. In CC models, curcumin significantly reduces the expression of VEGF, COX-2, and EGFR, thereby inhibiting tumor growth and angiogenesis [85]. Furthermore, in both subcutaneous and orthotopic non-small cell lung cancer models, curcumin suppresses tumor growth by inhibiting NF-κB activity and COX-2 expression [82]. In the TPA-induced skin tumor model, curcumin significantly reduced the production of 5(S)-, 8(S)-, 12(S)-, and 15(S)-HETE by inhibiting the activities of epidermal COX and LOX, and it blocked the metabolic conversion of arachidonic acid to PGE2, PGD2, and PGF2α [91]. Additionally, curcumin alleviated ear edema symptoms by inhibiting arachidonic acid metabolism [92]. In the pancreatic cancer animal model, the combination of curcumin and omega-3 fatty acids synergistically reduced the expression and activity of iNOS, COX-2, and 5-LOX in tumors, thereby inhibiting tumor progression [83]. Clinical trial results further confirmed that in patients with advanced pancreatic cancer, the expression levels of NF-κB, COX-2, and STAT3 in peripheral blood mononuclear cells (PBMCs) were significantly reduced following curcumin treatment [93].

#### 5.4.2. Indirect Effects on Arachidonic Acid Metabolism Through Regulation of Signaling Pathways

Curcumin can indirectly modulate arachidonic acid metabolism in tumor cells by targeting and regulating key signaling pathways, including NF-κB, AMPK, p38MAPK, and ERK. In head and neck cancer cells, curcumin activates AMPKα and p38MAPK signaling, enhances PGC-1α protein expression, and decreases Sp1 protein levels, thereby inhibiting the expression of the PGE2 receptor EP4 gene and effectively suppressing cancer cell proliferation [87]. In human breast epithelial cells, curcumin inhibits ERK1/2 and NF-κB transcriptional activity, reduces TPA-induced upregulation of COX-2 and MMP-9, decreases PGE2 synthesis, and counteracts tumor metastasis and invasion [78]. Furthermore, in human oral precancerous lesions and cancer cells, curcumin can inhibit the activation of NF-κB and COX-2 induced by the tobacco carcinogen 4-(methylnitrosamino)-1-(3-pyridyl)-1-butanone (NNK) [88]. In a mouse model of skin tumors, curcumin inhibits UVB radiation-induced inflammation and carcinogenesis by downregulating the expression of NF-κB, COX-2, PGE2, and NO [86]. Additionally, in melanoma and CRC cells, curcumin induces apoptosis by suppressing NF-κB activity and the expression of its downstream target genes, including COX-2, Bcl-2, Bcl-x(L), and Cyclin D1 [89,94]. In colon cancer cells, curcumin blocks cell proliferation and triggers apoptosis by activating the AMPK signaling pathway, which inhibits the expression of Akt and COX-2 proteins [73]. Moreover, in lung cancer cells, curcumin inhibits the biosynthesis of PGE2 by blocking IL-1β-induced expression of mPGES-1 and COX-2 through the suppression of EGR-1 expression, NF-κB, and JNK1/2 signaling [80]. These findings indicate that curcumin exerts its antitumor effects through multi-level and multi-target signaling regulation, indirectly influencing arachidonic acid metabolism, which represents a crucial mechanism of its action.

## 6. Curcumin Exerts Anti-Cancer Effects by Regulating Tumor Ferroptosis

Ferroptosis is an iron-dependent, non-apoptotic form of programmed cell death characterized by intracellular iron overload, depletion of glutathione (GSH), excessive accumulation of lipid ROS, and lipid peroxidation. The core mechanism of ferroptosis involves the iron-catalyzed peroxidation of polyunsaturated fatty acid phospholipids (PUFA-PLs), which surpasses the cell’s antioxidant defense threshold. This phenomenon primarily results from the inactivation or downregulation of GPX4, leading to the collapse of the GSH-dependent antioxidant defense system and the subsequent loss of the cell’s ability to eliminate lipid peroxides [95]. Additionally, the dysfunction of iron metabolism-related proteins, such as transferrin receptor 1 (TFR1), ferritin heavy chain (FTH1), and light chain (FTL), contributes to cellular iron overload, further exacerbating oxidative damage [96,97,98]. Curcumin can intervene in the aforementioned processes through multiple targets, including the upregulation of ACSL4 to promote the synthesis of polyunsaturated fatty acid-phosphatidylethanolamine (PUFA-PE), and the inhibition of SLC7A11 to limit GSH synthesis, thereby triggering the collapse of the antioxidant defense system [95]. Additionally, it can induce an imbalance in intracellular iron metabolism by regulating the expression of transferrin receptor 1 (TFR1), ferritin heavy chain 1 (FTH1), and ferritin light chain (FTL), and synergistically interfere with the activity of key molecules such as Nrf2 and GPX4. Ultimately, this disruption of intracellular redox homeostasis drives the ferroptosis program, exerting anti-tumor effects [96] (Figure 1, Table 4).

### 6.1. Mechanisms of Curcumin-Induced Ferroptosis in Different Tumor Types

Curcumin has shown potent ferroptosis-inducing capabilities across various malignant tumor models; however, the molecular mechanisms underlying these effects exhibit significant heterogeneity among different tumor types (Table 4). In the context of digestive system tumors, such as CRC, the mechanisms are diverse: in SW-480 cells, curcumin inhibits the JNK pathway, leading to GPX4 and FTH1 downregulation and ACSL4 upregulation. This results in significant accumulation of intracellular ROS, Fe^2+^, and lipid peroxides [117]. Concurrently, curcumin reshapes the ferroptosis-related gene network, characterized by the downregulation of MYC, IL-1B, and EZH2 mRNA expression and the upregulation of SLC1A5 and CAV1 expression, further enhancing the ferroptosis process [95]. In HCT-8 cells, curcumin significantly reduces the expression levels of GSH, SLC7A11, and GPX4 by inhibiting the PI3K/Akt/mTOR signaling pathway, while inducing the accumulation of ROS, malondialdehyde (MDA), and iron ions [5]. In SW620 and LoVo cell lines, as well as in the SW620 nude mouse xenograft model, curcumin significantly enhanced indicators characteristic of ferroptosis. These indicators include lactate dehydrogenase (LDH) release, ROS accumulation, lipid peroxidation, ferrous ion (Fe^2+^) accumulation, and malondialdehyde (MDA) levels. Concurrently, curcumin reduced GSH content and GPX4 activity by activating the p53 signaling pathway and inhibiting the SLC7A11/GSH/GPX4 axis [118]. In the GC AGS/HGC-27 cell model, curcumin activates the autophagy process by inhibiting the PI3K/Akt/mTOR signaling pathway, as evidenced by the upregulation of autophagy-related genes such as ATG5, ATG7, Beclin-1, and microtubule-associated protein 1 light chain 3B (LC3B). Concurrently, curcumin induces characteristic changes associated with ferroptosis, including a significant elevation in levels of ferrous ions (Fe^2+^), malondialdehyde (MDA), ROS, and ACSL4. This process is accompanied by a downregulation of SLC7A11, GPX4, and GSH levels [107]. Notably, Toll-like receptor 4 (TLR4) and KRAS signaling act as core regulatory modules of ferroptosis, and curcumin can specifically modulate these pathways to enhance the extent of ferroptosis [127].

In various solid tumors, the mechanisms through which curcumin induces ferroptosis exhibit significant diversity, primarily depending on specific cell lines or animal models. For example, in HCC cells and xenograft tumor models in nude mice, curcumin significantly downregulates the expression of SLC7A11, GPX4, GSS, and FTH1, while upregulating ACSL4 and PTGS2, thereby altering the ferroptosis-sensitive phenotype. This alteration results in the accumulation of Fe^2+^ and lipid peroxides (MDA), alongside the depletion of GSH, ultimately inhibiting tumor proliferation and growth [108]. In HCC cell lines PLC, KMCH, and Huh-7, curcumin induces ferroptosis by modulating genes associated with metal ion homeostasis, including CYP1A1, HMGCS2, HMOX1, LCN2, and MTTP. However, the KMCH and Huh-7 cell lines did not exhibit a significant ferroptosis phenotype due to subtype heterogeneity, indicating that HCC cells respond differentially to the ferroptosis-inducing effects of curcumin [109].

In BC, the mechanism of action is cell line-specific and involves multi-target regulation. Studies have demonstrated that in BC cell lines, specifically MCF-7 and MDA-MB-231, curcumin significantly upregulates the expression of ferritin light chain (FTL), ferritin heavy chain 1 (FTH1), and transferrin receptor (TFRC). Concurrently, it inhibits GPX4 and activates Nrf2 and heme oxygenase-1 (HO-1). This results in an abnormal accumulation of intracellular Fe^2+^, ROS, lipid peroxides, and malondialdehyde (MDA), along with a depletion of GSH [96]. Furthermore, curcumin can simultaneously activate oxidative stress pathways (HO-1, HSPA5), endoplasmic reticulum stress responses (DDIT3, ATF4), and transcriptional regulatory networks (BACH1, RELA, USF1, and NFE2L2), thereby amplifying the ferroptosis signaling cascade through the autophagy-redox pathway (GCLC, SQSTM1, XBP1, and BECN1) [96]. In MDA-MB-231 cells, curcumin significantly increased the levels of intracellular Fe^2+^, malondialdehyde (MDA), lipid hydroperoxide (LOOH), and ROS by promoting HO-1 hyperactivation, while simultaneously inhibiting the expression of ferritin heavy chain (FTH) and GPX4 and depleting GSH [100]. This cascade ultimately induced ferroptosis through an imbalance in Fe^2+^ homeostasis and collapse of the mitochondrial membrane potential. In MDA-MB-453/MCF-7 cells, curcumin promoted glutamine uptake by upregulating solute carrier family 1 member 5 (SLC1A5), synergistically inhibiting FTH and GPx4 expression, and activating ACSL4 and NADPH oxidase 1 (Nox1), which drove the accumulation of lipid ROS, MDA, and Fe^2+^. Animal experiments further confirmed that curcumin triggered GSH depletion and iron overload by promoting SLC1A5 overexpression in tumor tissues [101]. These findings demonstrate that the signaling network associated with curcumin-induced ferroptosis exhibits significant cell type dependency.

In non-small cell lung cancer (NSCLC), the underlying mechanisms are diverse. For example, in highly tumorigenic CD133^+^ A549 cells and xenograft models, curcumin significantly downregulates the expression of GPX4 and FSP1 proteins by targeting and inhibiting two key signaling pathways: GSH-GPX4 and FSP1-CoQ10-NADPH [110]. Additionally, in A549 and H1299 cells, as well as in animal models, curcumin exerts its anti-tumor effects by activating the autophagy-ferroptosis axis. This activation involves the upregulation of autophagy markers Beclin-1 and LC3-II, which facilitate autophagy, while also synergistically regulating key molecules associated with ferroptosis—specifically, the upregulation of ACSL4 and the downregulation of SLC7A11/GPX4 [112]. These processes lead to the accumulation of MDA, a decrease in SOD activity, depletion of GSH, and iron overload within tumor tissues. Functional validation indicates that the activation of autophagy is a crucial factor in inducing ferroptosis. In 16HBE, LK-2, and H1650 cells, as well as in animal models, curcumin targets and inhibits the DMRT3/SLC7A11 signaling pathway, which synergistically regulates key molecules involved in ferroptosis by downregulating GPX4 and upregulating ACSL4 and TFR1. This process induces a redox imbalance characterized by a significant reduction in SOD and GSH levels, along with an accumulation of MDA, LDH, and Fe^2+^ [113]. These findings suggest that the effects and pathways of curcumin-induced ferroptosis vary among different tumor types and even subtypes.

Furthermore, in acute B-lymphocytic leukemia cells, curcumin enhances the expression of ACSL4 while inhibiting the SLC7A11/GPX4 axis, resulting in GSH depletion, Fe^2+^ imbalance, and lipid peroxidation. The combined inhibition of this axis with human telomerase reverse transcriptase (hTERT) further synergistically promotes the activation of ferroptosis [99]. In thyroid follicular carcinoma (FTC-133/FTC-238 cells), curcumin induces intracellular iron accumulation, significantly increasing levels of malondialdehyde (MDA) and lipid ROS, while concomitantly depleting GSH [122]. This process is mediated by the aberrant activation of heme oxygenase-1 (HO-1) and the inhibition of GPX4 expression. In osteosarcoma MNNG/HOS/MG-63 cells and corresponding animal models, curcumin cooperatively regulates the Nrf2, SLC7A11, HO-1, and GPX4 signaling axis, resulting in abnormal accumulation of ROS and MDA, and the collapse of the GSH antioxidant system [123]. In a drug-resistant clear cell RCC model, curcumin induces ferroptosis and enhances sensitivity to sunitinib by upregulating ADAMTS18 gene expression, while downregulating the mRNA and protein expression levels of nuclear receptor coactivator 4 (NCOA4), ferritin heavy chain 1 (FTH1), and p53 [15].

Although curcumin has been reported to induce ferroptosis in various cancer types, its core signaling axis, such as the inhibition of SLC7A11/GPX4, exhibits significant variations across different tumor types. For instance, this process is often associated with p53 activation in CRC, while in BC, it may interact with the Nrf2/HO-1 pathway. This context-dependence suggests that the intrinsic genetic background and metabolic state of tumor cells determine their sensitivity to curcumin-induced ferroptosis and the specific response pathways involved. Therefore, when applying curcumin as a ferroptosis inducer, it is essential to consider the molecular subtype of tumors. Additionally, most evidence is derived from cell line studies, which have limitations in reflecting the complexity of the tumor microenvironment, including factors such as hypoxia and cell–cell interactions.

### 6.2. Synergistic Effects of Curcumin Combinations in Inducing Ferroptosis

Curcumin, when combined with various natural compounds or drugs, can synergistically induce ferroptosis in tumor cells through distinct molecular mechanisms. This demonstrates its potential value for combination therapy, which warrants further exploration in subsequent studies. In colon cancer models, the pairing of curcumin with andrographis activates ferroptosis through the dual inhibition of GPX-4 and FSP-1 expression [119]. Similarly, in CRC cells, the combination of curcumin and metformin significantly elevates lipid peroxidation levels by diminishing the activity of the xCT-GPX4 axis and downregulating DMT1 protein expression [120]. In lung cancer cells and animal models, the combination of curcumin and quercetin effectively advances the ferroptosis process by modulating the miR-520a-5p/SLC7A11 signaling pathway, which inhibits circFOXP1 expression, thereby suppressing tumor growth, migration, and invasion [111]. Collectively, these findings underscore the substantial therapeutic potential of curcumin combination therapy through multi-target regulation of the ferroptosis pathway.

### 6.3. Application and Advantages of Curcumin Derivatives in Inducing Ferroptosis

To address the inherent limitations of curcumin, such as low bioavailability and rapid metabolism, various derivatives have been developed through structural optimization. These derivatives demonstrate enhanced capabilities for inducing ferroptosis and improved targeting specificity. In ovarian cancer cells and animal models, the derivative NL01 effectively targets and inhibits the lactate metabolism receptors HCAR1/MCT1, thereby obstructing energy metabolism. This action subsequently activates AMPK, which synergistically downregulates the expression of SREBP1, SCD1, GPX4, and SLC11A2, ultimately leading to lipid peroxidation, disruption of energy metabolism, and the induction of ferroptosis [126]. In osteosarcoma cells, the derivative EF24 activates heme oxygenase 1 (HMOX1) in a dose-dependent manner while suppressing GPX4 expression, resulting in increased intracellular levels of MDA, ROS, and Fe^2+^ [124]. In non-small cell lung cancer, the derivative HO-3867 induces ROS and iron accumulation in a dose-dependent manner, mediating ferroptosis through the activation of the p53-DMT1 signaling pathway and the inhibition of GPX4 [114]. In BC cells, the derivative 4d specifically targets the SLC7A11/GPX4 signaling pathway, leading to an accumulation of Fe^2+^, increased levels of ROS and malondialdehyde (MDA), which collectively induce ferroptosis [105]. In glioblastoma cells, the derivative ALZ003 reduces GPX4 expression by promoting FBXL2-mediated ubiquitination of the androgen receptor (AR), resulting in significant lipid peroxidation and ROS accumulation. This mechanism not only induces ferroptosis in tumor cells but also enhances resistance to temozolomide [121]. In melanoma cells, the analog MitoCur-1 inhibits cystine uptake by targeting SLC7A11 and suppresses the deubiquitinating enzyme USP14. This action leads to the collapse of the antioxidant defense system involving GSH and GPX4, which synergistically promotes the abnormal accumulation of lipid ROS and Fe^2+^. Consequently, this activates the ferroptosis pathway and reverses resistance to vemurafenib [125]. In lung cancer cells, the derivative 2c triggers a significant generation of ROS and depletion of GSH by inhibiting TrxR activity, resulting in the inactivation of GPX4. This, in turn, induces intracellular lipid peroxidation and accumulation of malondialdehyde (MDA), leading to cell ferroptosis [115]. These studies systematically elucidate the multi-target synergistic mechanism of curcumin derivatives through the intervention of key iron metabolism hubs (Fe^2+^ accumulation), redox homeostasis (surge of ROS/MDA), and critical regulatory nodes (inhibition of GPX4/SLC7A11), thereby providing innovative strategies for the development of novel anti-cancer drugs aimed at enhancing ferroptosis.

### 6.4. Nano-Delivery Systems Enhance the Ferroptosis-Inducing Effect of Curcumin

In response to the inherent drawbacks of curcumin, such as poor bioavailability, inadequate absorption, and rapid metabolism, the development of novel intelligent nano-delivery systems has significantly enhanced its capacity to induce ferroptosis through spatiotemporal controlled release, microenvironmental responsiveness, and multimodal damage integration [128]. For instance, Liu et al. constructed a hypoxia-responsive nano-carrier, AAAF (AA/ASP-AZO-Fc), utilizing *Angelica sinensis* polysaccharide. Using curcumin as the model drug, AAAF@Cur micelles induced GSH depletion by consuming NADPH, thereby synergistically activating ferroptosis in tumor cells by enhancing ferritin sensitivity in a mouse model of liver cancer [127]. Additionally, the pH/ROS dual-responsive gold nanorods (Au NRs) designed by Zhong et al. can exploit the acidic pH and high ROS conditions characteristic of the tumor microenvironment to significantly induce the specific accumulation of lipid peroxides, thereby triggering ferroptosis in melanoma cells [129]. The calcium peroxide nanosystem developed by Yin et al. achieves tumor targeting through the co-loading of curcumin (CUR) and transferrin (Tf). This system can simultaneously release Ca^2+^ and curcumin in BC cells, resulting in Ca^2+^ overload that triggers the collapse of the mitochondrial membrane potential. The H_2_O_2_ generated from the reaction promotes the disintegration of the transferrin (Tf) structure, which synchronously releases Fe^3+^ to drive ferroptosis [102]. Fan et al. utilized cancer cell membrane-coated mesoporous silica nanoparticles (CM-MSN) loaded with curcumin (CM-MSN@CUR) to upregulate HO-1 and inhibit GPX4 in GC cell lines (SGC-7901, MGC-803) and xenograft tumor models. This approach led to lipid peroxidation (increased MDA levels), iron accumulation, ROS burst, and simultaneous GSH depletion, thereby inducing ferroptosis and inhibiting tumor growth [106]. Guo et al. utilized a copper-based metal–organic framework (MOF-199) to co-load curcumin and doxorubicin nanoparticles (Cur@DOX@MOF-199). In BC cells and animal models, they induced a significant generation of ROS by depleting GSH. This process subsequently activated the expression of heme oxygenase-1 (HO-1), which promoted the accumulation of Fe^2+^ and synergistically inhibited GPX4, leading to lipid peroxidation. Consequently, this triggered ferroptosis, resulting in a highly effective antitumor effect [103]. Xu et al. developed glucose oxidase (GOx)-modified ferritin nanoparticles loaded with luminol-curcumin. Upon targeted internalization into BC cells, these nanoparticles released a substantial amount of ROS, which not only induced apoptosis by collapsing the mitochondrial membrane potential but also activated the ferroptosis pathway by depleting reduced GSH and downregulating GPX4, thereby achieving an anti-tumor effect [104].

The development of intelligent nanosystems presents promising strategies and potential translational directions for systematically addressing the pharmacokinetic limitations of curcumin and enhancing its ability to induce ferroptosis; however, their safety and clinical efficacy necessitate further evaluation. It should be noted that most novel delivery systems discussed in this section are currently in the early to middle stages of preclinical development. The existing evidence primarily focuses on demonstrating the formulations’ ability to effectively enhance curcumin’s solubility, stability, targeting capability, and both in vitro and in vivo efficacy. However, systematic preclinical pharmacokinetic studies—such as comprehensive assessments of bioavailability, distribution, metabolism, and excretion—are generally lacking or insufficiently reported in these models. This absence of critical information represents a significant limitation in evaluating their true translational potential. Future studies must urgently incorporate standardized pharmacokinetic and pharmacodynamic (PK/PD) evaluations into the development pipeline.

## 7. Curcumin Regulates Tumor Amino Acid and Protein Metabolism

The malignant progression of tumors and the essence of their metabolic reprogramming are rooted in the dysfunction of a series of signaling molecules. Curcumin, characterized by its unique β-diketone structure and phenolic hydroxyl groups, can directly interact with multiple protein targets through intermolecular forces, including covalent modification and hydrophobic binding. This capability for direct binding enables curcumin to broadly regulate the activity of various functional proteins, such as metabolic enzymes, transcription factors, and protein kinases, thereby facilitating multidimensional intervention in tumor signaling networks [7,8]. Notably, curcumin’s ability to interact with multiple amino acid residues forms the molecular basis for its direct intervention in protein functions, subsequently influencing the metabolic reprogramming of tumor cells. This chapter will focus on elucidating how curcumin inhibits tumor progression through the precise regulation of amino acid metabolic networks and the processes of protein synthesis and degradation (Figure 1, Table 5).

### 7.1. Broad Regulation of Amino Acid Metabolism

The rapid proliferation of tumor cells is significantly reliant on the availability of amino acids, which serve as essential substrates for protein synthesis and energy metabolism [138]. Curcumin exerts its anti-tumor effects through multi-dimensional interventions in the amino acid metabolic network of tumor cells, with these interventions predominantly revealed in specific tumor models [139]. The mechanisms primarily involve regulating amino acid uptake, metabolic pathways, and key enzyme activities (Table 5). Regarding amino acid uptake, the curcumin analog WZ35 markedly decreases the uptake of glutamate and cysteine by downregulating the expression of SLC7A11 and SLC1A5 in GC cells, while also multi-dimensionally regulating GSH metabolism [14]. At the level of metabolic reprogramming regulation, WZ35 activates the ROS-YAP-AXL-ALKBH5-GLS2 signaling pathway, which induces and maintains a GSH-depleted cell phenotype. This is accomplished by reducing GSH precursors, including cysteine, glutamate, glycine, and glutamine; interrupting the regeneration of oxidized glutathione (GSSG); depleting GSH reserves due to the substantial generation of ROS; consuming the original GSH reserves; and accelerating the decomposition of GSH, with its decomposition product, cysteine, significantly accumulating. These processes synergistically reduce the levels of branched-chain amino acids and other amino acids, leading to the continuous consumption of metabolic substrates such as glutamate, cysteine, and leucine, ultimately inhibiting tumor proliferation, metastasis, and invasion [14]. In the BC model, curcumin exhibited specific regulation of the amino sugar metabolic axis, characterized by alterations in chitobiose, D-glucosamine-6-phosphate, L-fucose, and N-acetyl-β-mannosamine. Additionally, it influenced the amino acid biosynthesis metabolic pathway, evidenced by significant fluctuations in the levels of amino acids such as DL-isoleucine, DL-tyrosine, and homocysteine [130]. In CRC organoids, curcumin treatment led to substantial changes in the biosynthesis of phenylalanine, tyrosine, and tryptophan, as well as in the metabolic pathways of nicotinic acid and nicotinamide [131]. At the level of key metabolic enzyme targeting, curcumin inhibits the mRNA expression of BCAT1 in human myeloid leukemia cell lines and bone marrow mononuclear cells derived from acute myeloid leukemia (AML) patients. This inhibition reduces α-ketoglutarate (α-KG) levels, leading to defects in tumor cell growth and the induction of apoptosis [136]. In cholangiocarcinoma cells and animal models, curcumin suppresses the expression of SLC7A8 (LAT2) and works synergistically with gemcitabine to inhibit GLS and GS expression. This suppression reduces glutamine metabolism and ultimately induces cell apoptosis [137]. Additionally, in human promyelocytic leukemia cells, curcumin demonstrates pro-apoptotic effects by decreasing the activity and protein expression of ODC [140]. Collectively, these studies elucidate the molecular basis of curcumin’s anti-tumor effects by disrupting amino acid metabolic homeostasis, and also demonstrate that curcumin’s regulation of amino acid metabolism is context-dependent.

### 7.2. Intervention in Protein Metabolism

At the level of protein metabolism, curcumin influences tumor protein metabolism through its multi-target regulatory properties. Its mechanism of action involves key signaling pathways, including AMPK, MAPK, PI3K/Akt, NF-κB, and mTOR. For example, curcumin can directly inhibit mTOR activity, a core factor in regulating cellular growth and metabolism, reduce protein synthesis in tumor cells, and simultaneously activate autophagy and the ubiquitin-proteasome system to enhance protein degradation [141,142]. Curcumin has been shown to inhibit protein synthesis in tumor cells by modulating the activity of key proteins, including CDK2, CK2α, GSK3β, DYRK2, and EGFR [143]. Furthermore, curcumin can directly or indirectly inhibit the activity of crucial factors involved in the initiation of protein translation, such as eIF4E, thereby reducing the rate of protein synthesis in tumor cells [144]. In CRC cells and animal models, curcumin specifically downregulates the expression of the SIRT1 protein without affecting normal cells [141]. In leukemia cells, curcumin induces the formation of DNA topoisomerase complexes, specifically Topo I and Topo II-DNA complexes, which trigger apoptosis [145]. Proteomic studies have demonstrated that curcumin influences CRC cells by upregulating proteins associated with NAD/NADP metabolism, thereby enhancing redox metabolism. Concurrently, curcumin alters the expression of proteins related to RNA and lipid metabolism, such as ACOX1, ACSL1, HMGCS1, and PLIN3 [134]. Furthermore, it specifically upregulates the expression of proteins associated with copper toxicity stress, including MRPS14, GCLM, IMP4, and FAU, which mediates an imbalance in copper homeostasis and synergistically induces oxidative damage and cell death in tumor cells via the NADH/NADPH pathway [134]. These findings suggest that curcumin exerts its antitumor effects by selectively intervening in the reprogramming of amino acid and protein metabolism in tumor cells through the multi-target regulation of key enzyme activities.

### 7.3. In-Depth Regulation of Specific Amino Acid Metabolic Pathways

#### 7.3.1. Regulation of Tumor Glutamine Metabolism

Glutamine, recognized as the most abundant amino acid in the circulatory system, plays a central role in the metabolic reprogramming of tumors. Its metabolic rate is significantly elevated in tumor tissues. Upon uptake by cells via amino acid transporters such as ASCT2/SLC1A5 and LAT2, glutamine is deaminated by mitochondrial glutaminase (GLS), resulting in the production of glutamate. This process not only provides continuous nitrogen sources (e.g., for purine and pyrimidine synthesis) and carbon sources (through α-ketoglutarate entering the TCA cycle) essential for tumor proliferation but also maintains redox balance by generating precursors for glutathione, thus supporting oxidative stress defense during the rapid proliferation phase of tumors [146]. Given its critical role in metabolic pathways, targeting the regulation of glutamine metabolism has emerged as a vital strategy in anti-tumor therapy. Curcumin exerts anti-tumor effects by intervening in tumor glutamine metabolism through multiple mechanisms, including the inhibition of transporter function, interference with the activity of key enzymes, and disruption of metabolic homeostasis. These mechanisms have been validated in various tumor models [146].

In gemcitabine-resistant cholangiocarcinoma (CCA) cells, curcumin synergistically downregulates the expression of glutaminase (GLS) and glutamine synthetase (GS) by inhibiting LAT2-mediated glutamine (Gln) uptake in conjunction with gemcitabine. This mechanism leads to a reduction in glutamate (Glu) production and obstructs its involvement in critical metabolic processes, including nucleotide synthesis and the TCA cycle. Consequently, curcumin inhibits cell proliferation, induces cell cycle arrest, and promotes apoptosis, thereby ultimately overcoming chemotherapy resistance [137]. In vivo experiments further corroborated that the combination of curcumin and gemcitabine significantly decreases LAT2 levels in tumor tissues and inhibits the progression of xenograft tumors [137]. In colon cancer cells, curcumin reduces polyamine synthesis and D-glutamine metabolism by inhibiting the expression of ODC. It decreases levels of glutamate, GSH, and ATP, thereby inhibiting P-glycoprotein (P-gp) efflux activity. This action increases the intracellular accumulation of doxorubicin (Dox) and induces apoptosis, ultimately reversing tumor chemotherapeutic multidrug resistance (MDR) [4]. In CRC cells, curcumin treatment further reduces glutamine levels and promotes apoptosis [132]. In cisplatin-resistant CRC cells, curcumin disrupts glutamine metabolic homeostasis by inducing miR-137, which inhibits glutaminase (GLS) activity, thus restoring chemosensitivity to cisplatin [133]. In a prostate cancer model, curcumin, when combined with ursolic acid, targets ASCT2 to reduce glutamine uptake and inhibit tumor growth [135]. In adrenocortical carcinoma (ACC) cells, curcumin compels tumor cells to depend on glutamine metabolism for compensatory adaptation by upregulating the expression of SLC1A5 and GLS1. However, this glutamine-dependent adaptive reprogramming may inadvertently trigger ferroptosis through a ROS burst [46]. Similarly, in BC cells and animal models, curcumin enhances SLC1A5-dependent glutamine uptake, synergistically inhibits the iron storage protein FTH and the antioxidant enzyme GPX4, while activating ACSL4 and NOX1. This creates a multi-target ferroptosis-inducing network that significantly inhibits tumor growth [101]. Collectively, these studies reveal that curcumin disrupts tumor metabolic adaptability by intervening in glutamine metabolism, ultimately achieving tumor suppression and reversing drug resistance through mechanisms such as metabolic collapse, oxidative stress, and ferroptosis.

#### 7.3.2. Regulation of Tumor Polyamine Metabolism

Aberrant polyamine biosynthesis and elevated intracellular polyamine levels are universal biochemical characteristics observed across various cancer cell types, underscoring the critical role of polyamine homeostasis imbalance in tumorigenesis and progression [147]. Tumor cells induce dysregulated polyamine metabolism through mechanisms such as the upregulation of biosynthesis and enhanced polyamine uptake, which collectively promote proliferative dysregulation. As the key rate-limiting enzyme in polyamine synthesis, ODC catalyzes the conversion of L-ornithine to putrescine, representing the sole pathway for putrescine generation in mammals, and subsequently generates spermidine and spermine. Its activity is significantly induced by growth factors, hormones, and tumor promoters, such as 12-O-tetradecanoylphorbol-13-acetate (TPA), which directly regulate the DNA synthesis rate and proliferation processes in tumor cells. Studies have confirmed that, compared to normal tissues, the activity of ODC and levels of polyamines are significantly elevated in various human tumors, with their expression intensity showing a positive correlation with malignant tumor progression. Consequently, ODC is widely recognized as a tumor-promoting biomarker [147]. In this context, curcumin regulates polyamine metabolic homeostasis by targeting and inhibiting ODC activity, which not only disrupts tumor cell proliferation signals but also reverses metabolic reprogramming and induces apoptosis. This mechanism of action has been validated across multiple models, underscoring the potential of curcumin to exert anti-tumor effects by intervening in the polyamine pathway (Figure 1, Table 6).

The tumor promoter TPA has been shown to drive tumor progression by rapidly inducing ODC activity. In contrast, curcumin has exhibited antagonistic effects against TPA’s tumor-promoting actions across various experimental models. In vitro studies using mouse epidermal cells revealed that co-incubation with curcumin significantly inhibited the TPA-induced enhancement of ODC activity [156]. Furthermore, in a TPA-induced skin cancer animal model, the topical application of curcumin dose-dependently reduced both the elevation of epidermal ODC activity and skin tumorigenesis [92]. Lu et al. confirmed that curcumin could inhibit ODC activation in mouse skin epidermis by downregulating TPA-induced increases in ODC mRNA levels and accelerating its degradation [148]. Subsequent studies have demonstrated that the topical application of curcumin on the skin exerts antitumor effects by synergistically downregulating COX-2 and ODC activities, alleviating oxidative damage, and concurrently inhibiting inflammatory proliferation in the skin. This mechanism involves the inhibition of TPA-induced PKC translocation, activation of MAPK signaling pathways (JNK, ERK, and p38), and the expression of downstream transcription factors (c-jun and c-fos), ultimately leading to apoptosis [149]. Prolonged exposure to ultraviolet A (UVA) can synergistically enhance the induction of ODC activity and tumorigenesis in the skin of CD-1 mice. In contrast, curcumin treatment significantly mitigates the abnormal elevation of TPA-induced ODC activity, dermal inflammatory cell infiltration, and tumor progression [150]. Further mechanistic studies indicate that the synergistic activation of ODC and metallothionein (MT) by UVA and TPA is partially dependent on the ROS signaling pathway. Importantly, curcumin treatment effectively reverses this process, thereby blocking the combined tumor-promoting effects of UVA and TPA [151].

In BC cell lines, curcumin inhibits ODC activity and cell proliferation in a time- and dose-dependent manner [152]. Further studies indicate that curcumin regulates polyamine metabolism through the inhibition of the NF-κB signaling pathway, which triggers ROS-dependent cell cycle arrest and apoptosis. This effect is significant in both wild-type and Bcl-2 overexpressing drug-resistant MCF-7 cells [153]. In BC tumor spheroid models, curcumin reshapes the abnormally activated polyamine and lipid metabolism pathways by modulating metabolite levels and gene expression [60]. Additionally, in human promyelocytic leukemia cells, curcumin reduces ODC activity and protein expression in a dose- and time-dependent manner, inducing cell death by activating the ROS-dependent mitochondrial apoptosis pathway [140]. In drug-resistant colon cancer cells, curcumin inhibits polyamine synthesis and D-glutamine metabolism by suppressing the expression of ODC. This suppression leads to a reduction in intracellular levels of glutamate, GSH, and ATP, which results in diminished antioxidant capacity and inhibition of P-glycoprotein (P-gp) efflux function. Ultimately, this mechanism reverses multidrug resistance by increasing the intracellular accumulation of doxorubicin (Dox) and enhancing pro-apoptotic effects [4].

Moreover, curcumin has exhibited targeted inhibitory effects on ODC activity and associated metabolic pathways across various organ-specific carcinogenesis models. For example, in a rat model of colon cancer induced by azoxymethane (AOM), a diet supplemented with curcumin significantly reduced the activities of ODC, tyrosine protein kinase (TPK), and arachidonic acid metabolites in both the liver and colonic mucosa. This intervention also inhibited the formation of aberrant crypt foci (ACF), which are early hallmark lesions of colon cancer [74]. In a model of oral cancer induced by 4-nitroquinoline 1-oxide (4-NQO), curcumin treatment markedly suppressed tongue carcinogenesis by decreasing ODC activity and polyamine levels in the oral mucosa [154]. Furthermore, in a renal injury model induced by the nephrocarcinogen ferric nitrilotriacetate (Fe-NTA), dietary pretreatment with curcumin effectively mitigated renal oxidative stress, inhibited the abnormal increase in ODC activity, and alleviated renal histopathological damage [155].

## 8. Curcumin Regulates Tumor Mitochondrial Metabolism

### 8.1. Mitochondria and Apoptosis

Mitochondria, as dynamic organelles, are essential for cell survival, and their dysfunction can initiate programmed apoptosis through various mechanisms. This process is regulated by two primary pathways: the extrinsic pathway, activated by cell surface death receptors (such as FAS/CD95 and TNFR1/2), which leads to the activation of Caspase-8; and the intrinsic pathway, mediated by mitochondria, which depends on a cellular stress-induced imbalance in the Bcl-2 family. This imbalance results in the loss of mitochondrial membrane potential, the release of cytochrome c, and the formation of the apoptosome with Apoptotic Protease Activating Factor-1 (APAF-1), ultimately activating Caspase-9. Both pathways converge on the activation of effector Caspases-3, -6, and -7, which cleave PARP, inducing DNA fragmentation and culminating in apoptosis [157,158].

The mitochondrial permeability transition pore (mPTP) serves as a critical hub in apoptosis, comprising VDAC, ANT-1, and CyPD. Its opening results in the collapse of the membrane potential (mitochondrial depolarization), Ca^2+^ efflux, and the release of apoptotic proteins, including cytochrome c, Smac/DIABLO, AIF, and EndoG. This mechanism is particularly significant in curcumin-induced tumor cell death. The Bcl-2 family regulates the permeability of the outer mitochondrial membrane by maintaining a dynamic balance between pro-apoptotic proteins (Bax, Bid) and anti-apoptotic proteins (Bcl-2, Bcl-XL). Additionally, p53 can enhance mitochondrial apoptotic signaling by activating genes such as Noxa and PUMA. Furthermore, ROS levels influence cell fate through a biphasic effect: low concentrations activate the caspase cascade, while high concentrations induce necrosis, thereby accelerating the apoptotic process by disrupting redox homeostasis [157].

Curcumin and its analogs can activate mitochondrial-dependent apoptosis in various cancer cell types, including human glioblastoma, prostate cancer, lung cancer, and liver cancer. This process occurs through the modulation of the Bax/Bcl-2 ratio, leading to the loss of mitochondrial membrane potential and the subsequent release of apoptotic proteins. Consequently, a comprehensive apoptosis execution network is established, encompassing changes in membrane permeability and culminating in DNA fragmentation.

### 8.2. Curcumin Promotes Calcium Homeostasis Imbalance and Mitochondrial Membrane Potential Collapse to Induce Apoptosis

Curcumin triggers endoplasmic reticulum stress, leading to intracellular Ca^2+^ release. This disrupts mitochondrial calcium homeostasis and activates the mitochondrial permeability transition pore (mPTP). This series of events results in the loss of mitochondrial membrane potential (ΔΨm), which subsequently leads to mitochondrial swelling, cytochrome c efflux, and the activation of the caspase cascade (Figure 1). For instance, in human non-small cell lung cancer, GC, and colon cancer cells, curcumin induces endoplasmic reticulum stress, as evidenced by elevated levels of GADD153 and GRP78. It significantly increases Ca^2+^ levels, upregulates pro-apoptotic proteins BAX and BAD, and downregulates anti-apoptotic proteins Bcl-2, Bcl-XL, and XIAP. These changes culminate in the collapse of mitochondrial membrane potential and the leakage of cytochrome c into the cytoplasm, thereby activating the caspase-9/3 pathway and inducing apoptosis [159,160]. In papillary thyroid carcinoma cells, curcumin disrupts calcium homeostasis by inhibiting the activity of sarco/endoplasmic reticulum Ca^2+^-ATPase 2 (SERCA2). This inhibition leads to an increase in intracellular Ca^2+^ concentration and the formation of calcium/calmodulin complexes, which subsequently activate the CaMKII/JNK signaling pathway, thereby reducing mitochondrial membrane potential and triggering the apoptotic program. Concurrently, curcumin activates the endoplasmic reticulum stress response, promoting the generation of the spliced form of X-box binding protein 1 (XBP-1s) via the IRE1α-XBP-1s axis. It also upregulates the expression of endoplasmic reticulum-associated degradation genes such as ERDJ3, EDEM1, and SERP1, while synergistically enhancing the expression of the pro-apoptotic factor CHOP through the ATF-CHOP signaling pathway. Ultimately, curcumin induces apoptosis through the multidimensional regulation of calcium signaling dysregulation and endoplasmic reticulum stress [161].

In human tongue squamous cell carcinoma, curcumin treatment not only induces endoplasmic reticulum stress and mitochondrial dysfunction, but also facilitates the release of mitochondrial proteins such as AIF and Endo G, thereby establishing dual apoptotic pathways that are both caspase-dependent and caspase-independent [162]. A notable characteristic of tumor cells is the abnormal elevation of mitochondrial membrane potential. In comparison to normal cells, tumor cells exhibit resistance to apoptosis and promote metabolic reprogramming by sustaining a higher transmembrane potential. Curcumin can directly induce the loss of mitochondrial membrane potential in tumor cells by modulating the Bax/Bcl-2 ratio and inhibiting the ATP-sensitive potassium channel (mitoKATP) located on the mitochondrial membrane, consequently triggering mitochondria-dependent apoptosis [163].

In lymphoma cells, curcumin promotes an imbalance between Bax and Bcl-2, which is accompanied by a loss of mitochondrial membrane potential. This imbalance leads to the release of cytochrome c and the activation of the caspase-dependent apoptotic pathway [164]. The antitumor effects of curcumin have also been confirmed in models of various cancers, including human chronic and acute myeloid leukemia [76,165], lung cancer [166], CRC [167], gallbladder cancer [168], GC [169], liver cancer [170], retinoblastoma [171], and cervical cancer [172]. Furthermore, in osteosarcoma cells, curcumin enhances the permeability of the mitochondrial outer membrane by downregulating Bcl-2 and upregulating Bax, Bak, and p-Bad, thus inducing mitochondrial-dependent apoptosis [173]. In HCC cells and animal models, curcumin reduces mitochondrial membrane potential through BCLAF1-mediated PI3K/Akt/GSK-3β signaling, increases the levels of the apoptosis-related protein cytochrome c, and downregulates Bcl-2, ultimately executing the apoptosis program via Caspase-3 [174]. In human monocytic leukemia cells, curcumin not only induces a loss of mitochondrial membrane potential but also activates both intrinsic and extrinsic apoptosis pathways by stimulating JNK, p38MAPK, and ERK signaling [175]. These multi-target effects underscore curcumin’s capacity to integrate multiple signaling pathways through the collapse of mitochondrial membrane potential, thereby inducing mitochondrial-dependent apoptosis across various cancer types.

### 8.3. Curcumin Reshapes Tumor Energy Metabolism by Interfering with Mitochondrial Function

Curcumin has the potential to reshape tumor energy metabolism by interfering with mitochondrial function (Figure 1). In HNSCC and BC cells, curcumin demonstrates a dose-dependent inhibition of ATP synthase activity and induces the uncoupling of OXPHOS. This results in a significant decrease in both aerobic ATP synthesis and the OCR [176,177]. The metabolic inhibition exerted by curcumin is broad-spectrum; in leukemia, BC, melanoma, and colon cancer cells, it directly blocks ATP synthase activity in the mitochondrial membrane, leading to ATP depletion and respiratory suppression [142].

Further studies have demonstrated that in papillary thyroid carcinoma cells, curcumin aberrantly enhances succinate dehydrogenase (SDH) activity by binding to the SDHC subunit. This interaction triggers a burst of ROS and induces mitochondrial dysfunction, resulting in the collapse of the mitochondrial membrane potential, a reduction in the level of cytochrome c oxidase subunit II (COXII), and the loss of mitochondrial DNA (mtDNA), ultimately leading to excessive autophagy. In prostate cancer cells, curcumin decreases the production of acetyl-CoA, a substrate of the TCA cycle, by inhibiting PDH. This inhibition blocks the TCA cycle and reduces the activities of electron transport chain complexes I, III, and IV, exacerbating ATP depletion and ROS accumulation, and ultimately inducing both apoptosis and necroptosis in the cells [26].

In GC cells, curcumin promotes the generation of ROS, induces the depletion of DNA polymerase γ (POLG), and inhibits mtDNA replication. This cascade of events leads to a reduction in mtDNA content and downregulation of respiratory chain complexes, including COXI, COXII, COXIV, and CytB. Consequently, OXPHOS activity is suppressed, as evidenced by decreased OCR, ATP production, and maximal respiration, alongside reduced aerobic glycolysis, indicated by a diminished extracellular acidification rate (ECAR). This dual blockade effectively hampers the metabolic reprogramming associated with the Warburg effect in tumor cells. Ultimately, by disrupting mitochondrial homeostasis and inducing energy deprivation, curcumin synergistically inhibits the progression of GC [178]. In prostate cancer cells, curcumin improves the malignant metabolic phenotype by directly binding to pyruvate dehydrogenase (PDHA1), thereby reducing mitochondrial function [13].

It is noteworthy that curcumin can reverse tumor-associated metabolic disorders. In a mouse model of cachexia associated with triple-negative BC, curcumin alleviates mitochondrial dysfunction and increases ATP content in muscle tissue by regulating pyruvate metabolism, glycolysis/gluconeogenesis, and glyoxylate and dicarboxylate metabolism, while also improving metabolite profiles, including AMP, lysine, isobutyric acid, 3-hydroxyphenylacetic acid, and creatine. Concurrently, curcumin inhibits tumor proliferation and the progression of cachexia [179]. In GC cells, curcumin disrupts redox homeostasis by promoting ROS generation and reducing the activity of antioxidant proteins such as GPx1 and SOD2, which leads to mitochondrial dysfunction. This inhibition of OXPHOS and aerobic glycolysis results in a comprehensive decline in basal respiration, maximal respiration, glycolytic rate, and ATP production. Furthermore, the downregulation of Bcl-2 expression enhances the induction of mitochondrial-dependent apoptosis [45]. These effects underscore the potential of curcumin to exert tumor-suppressive effects by targeting mitochondrial metabolism.

### 8.4. Curcumin Induces Tumor Cell Apoptosis Through Oxidative Stress Regulation

Oxidative stress arises from an imbalance between the production and clearance of ROS, resulting in oxidative damage to DNA, proteins, and membrane structures. Mitochondria, as the primary site of ROS production, are particularly vulnerable to ROS-induced damage [1]. Curcumin exerts a dual effect through dose-dependent modulation of ROS levels. At low concentrations, tumor cells maintain redox balance by expelling oxidants, demonstrating reversible antiproliferative effects. Conversely, at high concentrations, curcumin causes a marked accumulation of ROS, surpassing the intracellular antioxidant threshold. This leads to mitochondrial dysfunction, collapse of mitochondrial membrane potential, release of cytochrome c, and activation of the caspase cascade. This process is further characterized by the upregulation of pro-apoptotic Bcl-2 family proteins (e.g., Bax) and the downregulation of anti-apoptotic members (e.g., Bcl-2), which enhances mitochondrial outer membrane permeability, resulting in the loss of membrane potential and the induction of apoptosis.

It is noteworthy that, compared to normal cells, tumor cells exhibit higher basal levels of ROS and lower activity of antioxidant enzymes, which renders them more susceptible to the pro-oxidant effects of curcumin. This susceptibility subsequently triggers depolarization of the mitochondrial membrane potential, leading to uncoupling of OXPHOS, depletion of ATP, and activation of mitophagy. Ultimately, this cascade results in programmed cell death through the activation of Caspase-3 and cleavage of PARP. In contrast, normal cells can tolerate such damage due to their stronger redox buffering capacity [1]. Therefore, a regulatory strategy that leverages this ‘threshold effect’ of oxidative stress enhances curcumin’s anti-tumor effects by targeting mitochondrial ROS metabolism.

Numerous studies have demonstrated that curcumin induces the generation of ROS in a dose-dependent manner, disrupts redox homeostasis within tumor cells, and activates the mitochondria-dependent apoptotic pathway. In various tumor types, including lymphoma [180], lung cancer [181,182,183], prostate cancer [184,185], non-small cell lung cancer [186], cholangiocarcinoma [187], melanoma [39,188,189], cutaneous T-cell lymphoma [190], and intestinal adenocarcinoma [191], curcumin triggers a significant accumulation of ROS, which leads to the collapse of the mitochondrial membrane potential. This collapse promotes the release of apoptotic factors such as cytochrome c, EndoG, Smac/DIABLO, and Omi/HtrA2 into the cytoplasm, thereby activating the Caspase-9/3 cascade and inducing apoptosis. This process is accompanied by a dynamic imbalance within the Bcl-2 family, characterized by the upregulation and translocation of pro-apoptotic proteins (Bax, Bad) to the mitochondria, while the expression of anti-apoptotic members (Bcl-2, Bcl-XL) is suppressed, further exacerbating the disruption of mitochondrial membrane permeability [181].

Curcumin compromises mitochondrial functional stability by inhibiting the activity of ODC in tumor cells, specifically in human acute promyelocytic leukemia cells [140,192]. It also reduces the levels of Hsp60 and HKII in human neuroblastoma cells, which synergizes with Ca^2+^ overload—observed in various cancers such as human thyroid cancer [193], leukemia [194], nasopharyngeal carcinoma [195], and lung cancer [181]—to further amplify oxidative damage. This cascade of events subsequently induces mitochondria-dependent apoptosis. Notably, curcumin exhibits similar pro-apoptotic effects on cancer-associated fibroblasts (CAFs), thereby inhibiting tumor progression through the ROS-endoplasmic reticulum stress-mitochondria-dependent apoptosis pathway [196].

### 8.5. Curcumin Analogs Target Mitochondrial Metabolism for Efficient Anti-Tumor Effects

Structurally optimized curcumin analogs enhance mitochondrial targeting, thereby improving antitumor efficacy and inducing tumor cell apoptosis through multiple synergistic mechanisms [197,198,199]. For example, MitoCur-1 selectively increases ROS generation in HCC cells by inhibiting thioredoxin reductase 2 (TrxR2), which simultaneously suppresses mitochondrial respiration and glycolytic metabolism, disrupting tumor metabolic plasticity [200]. WZ-26 promotes ROS accumulation in cholangiocarcinoma cells, resulting in the collapse of mitochondrial membrane potential and the activation of mitochondrial-dependent apoptosis [201]. In BC cells, WZ-35 induces mitochondrial dysfunction via the ROS-YAP-JNK signaling pathway, significantly inhibiting OXPHOS and reducing basal respiration, maximal respiration, spare respiratory capacity, and ATP production [202].

Structural optimization derivatives, including Bis-demethoxycurcumin (BDMC) for breast and gastric cancers [203,204], Tetrahydrocurcumin (THC) for BC [205], and Demethoxycurcumin (DMC) for lung cancer [193], can mediate alterations in mitochondrial outer membrane permeability by upregulating the Bax/Bcl-2 ratio and enhancing ROS accumulation. This process promotes the release of cytochrome c and activates the caspase-dependent apoptotic pathway [197,198,199]. Furthermore, EF24 (in CRC) [206], BDMC (in lung cancer) [207], WZ-35 (in prostate cancer) [208], WZ-37 (in HNSCC) [209], and 30-OH curcumin (in liver cancer) [210] exacerbate the depolarization of mitochondrial membrane potential through ROS-Ca^2+^ positive feedback signaling, which leads to mitochondrial dysfunction characterized by mitochondrial swelling, disruption of cristae structures, vesicular matrix swelling, loss of membrane potential, and release of cytochrome c, ultimately inducing apoptosis.

In oral cancer cells, the curcumin analog (PAC) inhibits the generation of ROS by enhancing GSH activity. It induces a collapse of the mitochondrial membrane potential (ΔΨm) and significantly increases the Bax/Bcl-2 protein ratio, which drives the release of cytochrome c. This process activates the Caspase-9/3-PARP-1 cleavage axis, ultimately triggering efficient cancer cell death through the mitochondrial-dependent apoptotic pathway [211]. These findings demonstrate the anti-tumor effects of targeting mitochondrial metabolism with curcumin analogs, providing a theoretical basis for the development of novel anti-cancer drugs.

## 9. Curcumin Exerts Anti-Tumor Effects by Regulating Gut Microbiota Metabolism

Curcumin exerts pleiotropic antitumor effects by targeting the gut microbiota-host metabolic axis. Despite its poor water solubility and low systemic bioavailability, curcumin effectively inhibits pro-carcinogenic microbiota, including toxin-producing and pro-inflammatory genera, while enriching probiotics such as *Lactobacillus* and *Bifidobacterium* in the gut. This reshaping of the microbiota structure enhances intestinal barrier function, reduces the accumulation of carcinogenic metabolites (such as branched-chain fatty acids and secondary bile acids), and reverses chemotherapy resistance [212]. This bidirectional regulatory mechanism effectively intervenes in gut microbial dysbiosis, which is closely associated with the development and progression of tumors, including CRC, BC, and liver cancer (Figure 1).

### 9.1. Efficacy of Curcumin in Reshaping Microbiota and Metabolism in Different Tumor Models

Curcumin has shown potential antitumor effects across various tumor models by modifying the structure of gut microbiota and its metabolic network. In CRC models, curcumin can mitigate the tumor-promoting effects induced by a high-protein diet (HPD) by decreasing levels of fecal ammonia, short-chain fatty acids (SCFAs), and branched-chain fatty acids (BCFAs), such as valeric acid and isovaleric acid. Additionally, it improves gut microbiota composition and reduces colon inflammation, cell proliferation, and the harmful luminal environment [213]. The study conducted by Deng et al. further corroborates that curcumin treatment can restore colorectal morphology and length in mice with colon cancer, inhibit tumor formation, and reshape the gut microbiota structure, resulting in significant restoration of both the diversity and richness of core and total microbiota. It notably reduces the abundance of pathogenic bacteria, including *Ileibacterium*, *Monoglobus*, and *Desulfovibrio*, while promoting the proliferation of beneficial bacteria such as *Clostridia_UCG-014*, *Bifidobacterium*, and *Lactobacillus* [2]. Metabolomic analysis revealed that the curcumin-treated group exhibited significant alterations in 13 differential metabolites. Notably, there was a reduction in the levels of procarcinogenic metabolites, such as ethionamide and xanthine, alongside an increase in protective metabolites, including glutamylleucine, γ-glutamylleucine, and rutin. These metabolites were primarily enriched in key pathways, including folate biosynthesis, purine metabolism, and ABC transporters. This suggests that curcumin may synergistically inhibit tumor progression by modulating the microbiota-metabolism axis [2]. In the colitis-associated CRC model, a curcumin-enriched diet reshapes colonic microbial ecology by significantly reducing the abundance of *Clostridiales* while promoting *Lactobacillales*, *Bifidobacteriales*, *Erysipelotrichales*, *Coriobacteriales*, and *Cyanobacteria YS2 microbiota*. Consequently, this intervention significantly reduces tumor burden, improves the colon weight/length ratio, and enhances survival rates [214].

In the HCC model, curcumin blocks the reprogramming of the tumor immune microenvironment (inhibiting M1 macrophage polarization and CD8^+^ T cell function) and ACC1-dependent fatty acid biosynthesis by the *B. thetaiotaomicron-acetate* metabolic axis through the inhibition of histone acetylation modification. This finding reveals curcumin’s ability to counteract the complex network of interactions between gut microbiota metabolites and the host by targeting epigenetic regulation, thereby reversing the dysregulation of the tumor immune microenvironment [215]. In studies of combination therapy, curcumin combined with 5-Fu significantly increased the abundance of gut microbiota in the H22 xenograft model of liver cancer, including *Helicobacteraceae*, *Campylobacterales*, *Helicobacter*, and *Campylobacter* within the *phylum Epsilonbacteraeota*, while specifically enriching *Bifidobacterium*, *Lactobacillus*, and *Escherichia-Shigella*. Additionally, experiments involving antibiotic-mediated gut microbiota depletion confirmed that the antitumor activity and chemosensitization effects of curcumin are entirely dependent on the regulation of gut microbiota [16].

Its efficacy has been validated in various tumor models. In an ulcerative CRC model, treatment with curcumin (CUR) and its aminated derivative (AC) significantly preserved colon length, reduced tumor numbers, and exerted protective effects by modulating the gut microbiota. Specifically, AC promoted the abundance of *Akkermansia*, *Bacteroides*, *Eubacterium fissicatena group*, *Candidatus Arthromilus*, *Ruminiclostridium*, and *Sphingomonas*. Both CUR and AC treatments upregulated the abundance of *Alistipes* while inhibiting the proliferation of *Candidatus Saccharimonas*, *Ruminiclostridium 6,* and *Ruminococcaceae UCG 005*, thereby delaying disease progression [216]. In an acute myeloid leukemia (AML) model, curcumin enhances the integrity of the intestinal barrier by enriching probiotics such as *Lactobacillus acidophilus*, *Bifidobacterium bifidum*, and *Lactobacillus reuteri*, while inhibiting pathogenic bacteria, including *Akkermansia muciniphila*, *Escherichia coli*, *Bacteroides fragilis*, and *Fusobacterium nucleatum*, thereby blocking bacterial and metabolite translocation. Additionally, it targets and inhibits the key enzyme squalene epoxidase (SQLE) in cholesterol biosynthesis, reducing cholesterol accumulation in mononuclear cells (MNCs) and ultimately synergistically reversing cytarabine (Ara-C) chemotherapy resistance [217]. These results indicate that curcumin exerts synergistic antitumor effects by reshaping the balance of gut microbiota and regulating the metabolic network.

### 9.2. Synergistic Regulation of Microbiota by Curcumin Formulations Based on Delivery Systems

To enhance the biological activity of curcumin, numerous studies have developed innovative delivery systems aimed at synergistically regulating gut microbiota and improving anti-tumor efficacy. For example, Zhang et al. constructed curcumin-modified selenium nanoparticles (Cur/Se), which not only significantly inhibited tumor growth in an ascites cancer mouse model but also reduced the abundance of harmful bacteria, such as *Rikenellaceae_RC9_gut_group*, *Enterorhabdus*, and *Bilophila*, while simultaneously increasing the levels of beneficial bacteria, including *Lachnospiraceae_UCG-006* and *Lactobacillus citreum*. Furthermore, Cur/Se exhibited a markedly superior ability to promote the proliferation of *Lactobacillus citreum* compared to curcumin alone, suggesting that Cur/Se enhances the bioactivity of curcumin by further reinforcing the regulation of gut microbiota [218].

The Zn(II)-curcumin solid dispersion (ZnCM-SD) developed by Wu et al. exerts synergistic effects through a dual mechanism in a rat model of HCC. Firstly, it significantly reduces the total bacterial load in feces and upregulates the abundance of *Bacteroidetes*, including *Barnesiella*, *Unclassified_Porphyromonadaceae*, *Paraprevotella*, and *Prevotella*, while simultaneously suppressing the abundance of *Firmicutes*, such as *unclassified Lachnospiraceae*, *Clostridium cluster XIVa*, *Pseudoflavonifractor*, and *Oscillibacter*. This alteration results in a decreased *Firmicutes/Bacteroidetes* (F/B) ratio, which serves as a marker of gut dysbiosis, effectively reversing intestinal dysbiosis [219]. Secondly, ZnCM-SD enhances intestinal barrier function by promoting villus growth, reducing crypt depth, increasing the number of goblet cells, upregulating the expression of ZO-1 and occludin, and improving intestinal permeability. Furthermore, fecal microbiota transplantation and microbiota depletion experiments confirm that its combination with doxorubicin can mitigate HCC-related gut microbiota disorders through microbiota remodeling [219].

In animal models of CRC, the combined intervention of Essential Turmeric Oil-Curcumin (ETO-Cur) and the tocotrienol-rich fraction (TRF) significantly increased the abundance of *Proteobacteria*, *Actinobacteria*, and beneficial bacterial groups, including *Porphyromonadaceae*, *Lactobacillaceae*, *Rickenellaceae*, *Desulfovibrionaceae*, *Enterobacteriaceae*, *Bifidobacteriaceae*, and *Firmicutes*. This intervention inhibited the growth of *Bacteroidaceae*, which includes *Lachnospiraceae* and *Ruminococcaceae*, while specifically enriching probiotic families such as *Clostridium IV* and anti-inflammatory *Lactobacillaceae*. Consequently, this reshaped the gut microbiota structure, facilitating tumor suppression [212]. Collectively, these studies indicate that curcuminoids, through the optimization of delivery systems, provide a significant foundation for tumor treatment strategies based on the regulation of the ‘microbiota-gut barrier’ axis.

## 10. Clinical Evidence for Curcumin’s Regulation of Tumor Metabolism

Preliminary clinical studies have provided evidence that curcumin modulates tumor metabolism and exerts antitumor effects. However, its clinical application is primarily constrained by pharmacokinetic challenges, such as low oral bioavailability and rapid in vivo metabolism. Additionally, most related clinical studies are still in the early phases (I/II) and involve limited sample sizes (Table 7). Phase I clinical trials indicate that curcumin demonstrates good tolerability, even at doses as high as 12 g/day; however, its clinical application is primarily constrained by the challenge of low oral bioavailability [220,221]. Despite this limitation, multiple studies have identified its potential efficacy and mechanisms of action in specific tumor types.

In the field of gastrointestinal tumors, particularly CRC, the research evidence supporting the efficacy of curcumin is the most substantial. Clinical studies have demonstrated that daily doses of 2 g or 4 g of curcumin taken for one consecutive month can significantly reduce the formation of colorectal precancerous lesions, specifically ACF [222]. Furthermore, a dosage of 3.6g per day not only decreases the levels of inducible PGE2 in the bloodstream [223] and oxidative DNA adducts (M1G) in malignant tissues [223] but also achieves effective pharmacological concentrations in colorectal tissues [223,224]. Additionally, curcumin enhances intratumoral p53 expression, which promotes apoptosis and improves the overall health status of patients [224]. In vitro studies further corroborate its capacity to inhibit cancer stem cell (CSC)-like phenotypes and ALDH enzyme activity in CRC liver metastases [225]. Curcumin has shown promise in regulating metabolism in precancerous lesions and gliomas. High-risk patients with precancerous lesions exhibited no toxicity and demonstrated potential for cancer prevention after a daily intake of 8 g for three consecutive months [226]. In glioma patients, the oral administration of 70 mg curcumin capsules over four days resulted in a decreased phosphocreatine/inorganic phosphate ratio and an increased pH value within the tumors. These findings suggest that the therapeutic efficacy of curcumin may be mediated through the modulation of intratumoral energy metabolism [227].

Curcumin exhibits multifaceted effects in various solid tumors and supportive care. In patients with BC, administering curcumin leads to the accumulation of its metabolite in malignant breast tissue, which induces cell cycle arrest and apoptosis in p53-WT MCF-7 cells via the p53/p21 signaling pathway [228]. Prostate cancer patients who consumed 3g/day of curcumin for three consecutive months demonstrated an increase in serum total antioxidant capacity (TAC) and a decrease in superoxide dismutase (SOD) levels [229]. In pancreatic cancer patients, an intake of 8 g/day of curcumin for two months resulted in a significant downregulation of pro-inflammatory and pro-survival signals, including NF-κB, COX-2, and STAT3, in peripheral blood mononuclear cells (PBMCs) [93]. Furthermore, curcumin shows potential in the management of oral cancer and cancer cachexia. Oral administration of APG-157, a polyphenolic botanical drug containing curcumin, has been shown to reduce salivary inflammatory cytokines, modulate oral microbiota, and promote the recruitment of anti-tumor T cells [230]. Furthermore, a daily intake of 1.6 g of curcumin over eight consecutive weeks decreases the basal metabolic rate in patients suffering from cancer-associated cachexia syndrome (CACS), mitigates the decline in serum albumin levels, and delays the loss of grip strength as well as the progression of cachexia [231].

Although the aforementioned studies provide preliminary signals, a significant gap and fundamental limitations persist in the current chain of evidence regarding the clinical efficacy of curcumin in modulating tumor metabolism. While existing clinical evidence suggests potential activity, there remains a notable disparity in demonstrating curcumin’s antitumor effects through metabolic regulation, primarily constrained by the following fundamental issues: a direct lack of key mechanistic evidence. The vast majority of trials focus on traditional safety or imaging endpoints, with very few studies employing tumor-specific metabolic imaging (such as novel PET probes) or systemic metabolomic changes as core endpoints. This limitation complicates the ability to directly correlate human effects with the precise metabolic mechanisms elucidated in preclinical studies. Additionally, the pharmacokinetic bottleneck remains unaddressed. The inherent low bioavailability and rapid metabolism of curcumin hinder the achievement of effective concentrations at tumor sites following conventional administration. This leads to a disconnect between ‘clinical dosage’ and ‘mechanistically effective concentration,’ which is a core reason for translational failure. Furthermore, the strength of the evidence itself is limited. Most existing positive results originate from small-sample, early-phase single-arm studies, and the heterogeneity in curcumin formulations and patient populations raises questions about the reliability and generalizability of the evidence, necessitating validation through large-scale randomized controlled trials. Consequently, the current clinical evidence system remains incomplete, and the clinical translation of curcumin’s metabolic regulatory effects faces significant challenges. In summary, while most clinical studies are still in the early stages, the existing evidence demonstrates potential clinical value (Table 7).

**Table 7:** Clinical Evidence of Curcumin in Regulating Cancer Metabolism.

Tumor Type	Dose of Curcumin	Treatment Duration	Design	Regulatory Ways	Main Findings	Phase	Sample Size	Year	Refs
GBM	Curcumin (172.2 mg/d), demethoxycurcumin (33.6 mg/d), bis-demethoxycurcumin (4.2 mg/d)	4 days	During the surgical procedure, tumor and blood samples were collected for the analysis of total curcumin concentration. P magnetic resonance spectroscopic imaging was performed before and after curcuminoid consumption.	Energy metabolism	1—curcumin capsule treatment may change the intratumoral energy metabolism, in which the average ratio of creatine phosphate to inorganic phosphate is decreased, while the average intratumoral pH is increased 2—the total curcumin concentration in glioblastoma was quantifiable	Not specified	10	2016	[227]
CRC	3.6 g/d	30 days	Blood samples were collected at 1 h post-dose (Day 1 and Day 29) for analysis of glutathione S-transferase activity, M(1)G levels, and PGE(2) levels.	AA metabolism	1—taking 3.6 g curcumin per day can achieve pharmacologically effective levels in colorectal 2—decreased levels of inducible PGE2 in blood	Not specified	15	2004	[223]
CRC	1.8, 3.6, 4.5 mg/d	7 days	Blood samples were collected 1 h after the last administration. Curcumin and its metabolites were quantitatively analyzed using high-performance liquid chromatography (HPLC), ultraviolet spectrophotometry, and mass spectrometry.	Redox metabolism	the level of oxidized DNA adducts M1G was significantly reduced in malignant colorectal tissues	Phase I	12	2005	[232]
PC	3 g/d	3 months	Plasma total antioxidant capacity (TAC), superoxide dismutase (SOD), catalase, and glutathione peroxidase (GPx) activities were measured at baseline and 3 months after radiotherapy completion.	Redox metabolism	1—compared with baseline, patients taking curcumin had significantly higher TAC and lower SOD activity in serum 2—compared with the placebo group, the serum TAC of patients taking curcumin was significantly increased, while SOD was significantly decreased	Not specified	20	2016	[229]
Pancreatic cancer	8 g/d	2 months	Monitor serum levels of cytokines including interleukin (IL)-6, IL-8, IL-10 and IL-1 receptor antagonist, as well as the expression of NF-kappaB and cyclooxygenase-2 in peripheral blood mononuclear cells.	AA metabolism	the levels of NF-kB, COX-2 and STAT3 in PBMCs of patients were significantly down regulated	Phase II	25	2008	[93]
CACS in Solid Cancer	1600 mg/d	8 weeks	The primary endpoint was improvement in body composition, while the secondary endpoints included body weight and body mass index, enhancement of handgrip muscle strength, and safety.	Energy metabolism	1—decrease in basic metabolic rate and decrease in serum albumin level 2—produce clinical benefits in slowing grip strength loss and CACS progression	Phase IIa	33	2022	[231]

## 11. Summary and Prospects

This review systematically elaborates on the core mechanisms through which curcumin inhibits tumor progression by targeting metabolic reprogramming, a fundamental hallmark of cancer. Curcumin exerts broad-spectrum antitumor effects by precisely intervening in the glucose metabolism of tumor cells, notably by suppressing key glycolytic enzymes such as HK2, PKM2, and LDHA, as well as GLUT1 and MCTs, thereby effectively reversing the Warburg effect. In the realm of lipid metabolism, curcumin downregulates the expression and activity of central lipogenic enzymes, including FASN and SCD1, while simultaneously inducing ferroptosis by modulating the SLC7A11/GPX4 axis and upregulating ACSL4.

Furthermore, curcumin profoundly disrupts amino acid and protein metabolic networks. It impedes glutamine utilization by targeting GLS, disrupts polyamine homeostasis by inhibiting ODC, and interferes with protein synthesis and degradation processes. At the level of mitochondrial energy metabolism, curcumin impairs OXPHOS, promotes the collapse of the mitochondrial membrane potential, induces massive generation of ROS, and activates the mitochondrial apoptotic pathway. Beyond direct targeting of tumor cells, curcumin also reshapes the tumor microenvironment by modulating the gut microbiota-host metabolic axis. It enriches beneficial bacteria such as Lactobacillus and Bifidobacterium, suppresses pathogenic bacteria, and regulates microbial metabolites like short-chain fatty acids and bile acids, thereby enhancing intestinal barrier function and reversing chemotherapy resistance.

The multifaceted metabolic interventions of curcumin are inseparable from its synergistic regulation of pivotal signaling pathways. It inhibits pro-tumorigenic signals such as PI3K/Akt/mTOR and NF-κB, while activating tumor-suppressive pathways like AMPK, forming an intricate regulatory network that highlights its unique advantages and tremendous potential as a natural multi-target metabolic modulator.

Despite the promising preclinical findings, the translation of curcumin into widespread clinical practice encounters significant challenges. The primary issue is the inherent pharmacokinetic deficiencies associated with curcumin. Future research should prioritize the following areas: (1) employing multi-omics technologies to thoroughly elucidate its metabolic regulation and compensatory mechanisms; (2) enhancing combination strategies with conventional therapies and targeted metabolic therapies to mitigate drug resistance; (3) developing personalized treatment regimens based on biomarkers; and most critically, (4) vigorously advancing the creation of novel delivery systems, such as nano-formulations, and structurally optimized derivatives, while systematically assessing their pharmacokinetic improvements and definitive anti-tumor efficacy through well-designed, large-scale clinical trials to facilitate its clinical translation. Furthermore, we advocate for future studies to validate key findings across multiple cell lines, organoids, or in vivo models to address the limitations of single-model systems and bolster the reliability of conclusions.

At the basic research level, there is an urgent need to establish standardized in vitro experimental systems to elucidate the core metabolic effects of curcumin. This effort should include clarifying the interference effects of solvents, such as DMSO, implementing rigorous controls, promoting more physiologically relevant drug delivery methods, and integrating data on metabolic enzyme expression and activity with stable isotope tracing-based metabolic flux analysis. These approaches will move beyond static ‘snapshots’ to dynamically and precisely delineate the metabolic reprogramming landscape under curcumin intervention.

In conclusion, curcumin provides a solid theoretical foundation and abundant inspiration for developing innovative anti-tumor strategies centered on metabolic reprogramming. Its ability to orchestrate multi-dimensional regulation of the tumor metabolic network positions it as a valuable asset in the oncotherapeutic arsenal. Through sustained interdisciplinary collaboration, encompassing mechanistic exploration, formulation innovation, and clinical translation, curcumin is poised to be revitalized and integrated as a key component of future precision oncology paradigms.

## Figures and Tables

**Table 1:** The Regulatory Effect of Curcumin on Tumor Glucose Metabolism.

Compound	Disease	Type of Cell Lines or Animal Model	Regulatory Ways	Main Findings	Refs
Curcumin	Pancreatic cancer	Panc-1, SW1990	Glycolysis	1—inhibited Beclin1 and HIF-1α expression 2—reduce the activities of GLUT1, HK2, LDHA, PDK1 and ATP production 3—inhibition of cell proliferation	[20]
		BxPC-3	Glucose metabolism	1—inhibit EGF/EGFR and downstream ERK and Akt signals 2—inhibit hyperglycemia-driven cell invasion and migration	[22]
Curcumin	RCC	ACHN	Glycolysis	1—up regulate miRNA Let-7c to inhibit HIF-1α and PDK1 2—inhibiting glycolysis and reversing treatment resistance	[21]
Curcumin	CRC	HCT116, HT29	Glycolysis, Serin Pathway, PPP, Mitochondrial metabolism	1—down-regulation of HK2 inhibits glycolysis 2—activated mitochondrial apoptotic pathway	[12,23]
				Inhibit the activities of a variety of metabolic enzymes, including GAPDH, PKM1/2, LDHA, MDH1/2, PGD, PHGDH, TPI, PGK1, and ALDOA.	
				decreased glucose uptake and increased lactate production	[24]
Curcumin	PC	PC-3, DU145, mouse model	Glycolysis, Mitochondrial metabolism, Ferroptosis, TCA cycle	1—inhibits glycolysis phenotype, HK2 activity, and ATP levels 2—decreased respiratory chain complex I-V activity, increased ROS generation, and DNA damage trigger mitochondrial dysfunction	[25]
				1—inhibits ERK1/2-c-myc-HIF-1α signaling, reduces HK1, HK2, and PFKP activities, and blocks glycolysis metabolic flux and glucose consumption 2—decreased PDH activity, inhibited the TCA cycle, and acetyl-CoA production 3—the activities of ETC complexes (I, III, and IV) were decreased, and ATP depletion triggered cellular apoptosis and necroptosis	[26]
				1—inhibiting mTOR/HIF-1α downregulates PKM2 and reduces Warburg effect 2—inhibit GLUT1 and HK2 activities, reduce glucose uptake and lactate release	[17]
Curcumin	HCC	Huh7, Li-7, HepG2, HCCLM3, mouse model	Glycolysis	inhibition of CSN5 decreased HK2 expression and decreased glycolysis	[27]
		HepG2, HuT78	Glycolysis, TCA cycle	1—inhibited the expression of HIF-1α, IDH-α, GLUT1, MCT-1, MCT-4, MDR-1, HCAR-1 and STAT3 2—glucose consumption, lactate production, extracellular acidity, ROS, and NO levels were inhibited	[28,29]
				1—reduce glucose consumption, lactate efflux, and extracellular acidification 2—reduce NO and ROS production 3—inhibits the expression of metabolic enzymes (HK2, PFK1, GAPDH, PKM2, LDHA, IDH3A, and FASN) and related transporters (GLUT1, MCT1, MCT4, HCAR-1, and MDR1) 4—enhance SDH activity 5—inhibits HIF-1α, mTOR, Myc, and STAT3 activity	[30]
Curcumin	BC	MDA-MB-231, MCF-7, mouse model	Glycolysis, PPP, TCA cycle, OXPHOS	inhibit slug and HK2, reduce glycolysis, and mediate mitochondrial apoptosis	[31]
				1—inhibiting mTOR/HIF-1α downregulates PKM2 and reduces Warburg effect 2—inhibits GLUT1 and HK2 activities, reduces glucose uptake and lactate release	[17]
				inhibition of GLUT1 expression by PPARδ /Akt signaling	[32,33]
				inhibition of glycolysis and pentose phosphate metabolism triggers tumor metabolic compensation, resulting in upregulation of TCA cycle and OXPHOS pathway-related enzyme activities	[34,35]
Curcumin analog, 1,5-bis(4-Hydroxy-3-Methyoxyphenyl)-1,4-Pentadiene-3-One	GBM, NBL	U-87, SH-SY5Y	Glycolysis	inhibition of ENO1, GAPDH, TPI1, PGK1 activities reduces glycolysis	[36]
Curcumin	HNC	H157, H413	Glycolysis	inhibiting DNMT3b remodels PKM splicing, promotes the conversion of PKM2 to PKM1, and reduces the Warburg effect	[37]
Curcumin analog, GO-Y030	Melanoma	B16-F10	Glycolysis	inhibit GLUT1, GLUT4, PDK1, and PFKM, and reduce glucose uptake and glycolysis	[38]
Curcumin		A375	PPP, Mitochondrial metabolism	promotes ROS generation, reduces MMP, GSH, and G6PD activities, and inhibits cell proliferation	[39]
Curcumin	CML	K562	Glycolysis, PPP	1—inhibiting HIF-1α activity through miR-22/IPO7/HIF-1α axis 2—downregulate the activities of glycolysis enzymes (TPI1, GPI, MDH1, PGK1, GOT1), PPP enzymes (G6PD, PGD) and HIF-1α target genes (ALDOA, PKM, LDHA, and PGK1)	[40]
Curcumin	DL	mouse model	Glycolysis	targeting c-Myc and HIF-1α signals downregulate LDHA activity and expression, reduce glycolysis, and inhibit tumor progression	[41]
Curcumin	ESCC	EC109	Glycolysis	AMPK dependent downregulation of GLUT4, HK2, PFKFB3, PKM2 expression	[42]
Curcumin	PTC	B-CPAP	Glycolysis	inhibition of LDHA and HK2 reduces glucose uptake and lactate production, triggering apoptosis and Warburg effect inhibition	[43]
Curcumin analog, WZ35	GC	BGC-823, SGC-7901	Glycolysis, Mitochondrial metabolism	activating ROS/YAP/JNK signaling inhibits glycolysis	[44]
Curcumin				promote ROS generation, induce mitochondrial dysfunction, and downregulate OXPHOS and glycolysis activities	[45]
Curcumin	HNSCC	FaDu, Detroit562, Cal27	Glycolysis	inhibition of glucose uptake and lactate production	[24]
Curcumin	ACC	H295R, SW13, MUC-1	Glycolysis, TCA cycle	1—glycolysis, TCA cycle flux, and metabolic reserve capacity are limited 2—inhibit LDHA, ECAR, and OCR	[46]
Curcumin	CC	HeLa	Glycolysis	1—inhibiting mTOR/HIF-1α downregulates PKM2 and reduces Warburg effect 2—inhibits GLUT1 and HK2 activities, reduces glucose uptake and lactate release	[17]
Curcumin	Lung cancer	A549	Glycolysis	inhibition of GLUT1/MT1-MMP/MMP2 signaling inhibits cell invasion and metastasis	[47]
		H1299	Glycolysis	1—inhibiting mTOR/HIF-1α downregulates PKM2 and reduces Warburg effect 2—inhibits GLUT1 and HK2 activities, reduces glucose uptake and lactate release	[17]

**Table 2:** The regulatory effect of Curcumin on tumor lipid metabolism.

Compound	Disease	Type of Cell Lines or Animal Model	Regulatory Ways	Main Findings	Refs
Curcumin	HCC	HepG2	Lipid metabolism	1—activated AMPK upregulates PPARα and downregulates SREBP-1c and target gene FAS expression 2—inhibits lipid synthesis and promotes lipolysis, reducing TG and TC accumulation	[51]
				1—downregulates SLC13A5 and ACLY expression through AMPK mTOR signaling 2—reduced DNL and lipid accumulation	[52]
				inhibition of NPC1L1, SREBP-2 and HNF1α expression reduces cholesterol absorption	[53]
				1—promote the expression of LDLR, HMG-CoA reductase, FDPS, SREBP-2, LXR α, and target gene ABCG 1 2—down regulates CD36 and FABP1, and reduces cholesterol	[54]
				inhibit FAS activity and expression, induce apoptosis	[55]
		Huh-7	Lipid metabolism	1—downregulated the expression of SREBP and target genes, including genes related to fatty acid and triglyceride synthesis (SREBP-1, FAS, ACC1, SCD-1, SCD-2, ACL, FADS1, FADS2, and GPAT) and cholesterol synthesis (SREBP2, HMGCR, HMGCS, LSS, SC4MOL, SE, DHCR24, DHCR7, FDPS, MVK, and FDFT1) 2—block fatty acid and cholesterol anabolism	[56]
		mouse model	Lipid metabolism, Glycolysis	1—reduce the levels of serum LDH, TG, FASN, D-fructose, D-glucose, and lactate 2—upregulated HDL-C and APOAI mRNA levels 3—inhibited glycolysis (IDH/HIF-1α activity inhibition) and lipid anabolism (decreased levels of hexadecanoic acid and FASN)	[57]
Curcumin	PC	PC-3, DU145, LNCaP	Lipid metabolism	inhibition of SREBP-1/2, LDLR, M-CIC, HMGCR, and GPAT expression	[13]
Demethoxycurcumin				1—upregulate AMPK and reduce FASN expression 2—inhibition of ACC reduces lipid synthesis and intracellular total fatty acid content	[58]
Curcumin	BC	MDA-MB-231	Lipid metabolism	inhibit Fas activity and expression, induce apoptosis	[59]
				inhibit the expression of lipid metabolism-related genes FASN, SCD, ELOVL1, GPAM, CEPT1, and PTDSS1, and reduce fatty acid synthesis	[60]
		MDA-MB-453, MCF7	Lipid metabolism	inhibit the expression of cholesterol biosynthetic genes, including ELOV6, CYP51, LSS, MVD, SQLE, and FASN	[61]
Demethoxycurcumin		MDA-MB-231	Lipid metabolism	1—upregulates AMPK and reduces FASN and ACC expression 2—decreased intracellular lipid synthesis and total fatty acid content	[62]
Curcumin analog, GO-Y030 and GO-Y078	MM	RPMI8226, KMS12-BM, U266, OPM2	Lipid metabolism	inhibit SCD, NF-kB, PI3K/Akt, JAK/STAT3, and IRF4 signaling	[63]
Curcumin	Lung cancer	A549	Lipid metabolism	activate AMPK and its downstream ACC phosphorylation to induce autophagy	[64]
Curcumin	CRC	Caco-2	Lipid metabolism	inhibition of NPC1L1, SREBP-2 and HNF1 α expression reduces cholesterol uptake	[53,65]
				1—activate TRPA1 channels to stimulate Ca^2+^ influx 2—reduce cholesterol absorption through PPARγ /SP-1/SREBP-2/NPC1L1 cascade and synergistically inhibit cell proliferation	[66]
		HT29	Lipid metabolism	inhibit the expression of cholesterol biosynthetic genes, including ELOV6, CYP51, LSS, MVD, SQLE, and FASN	[61]
		mouse model	Lipid metabolism	1—inhibits CAMP/PKA/CREB signaling in ewat and downregulates the expression of key lipolytic proteins HSL, ATGL, and UCP1 2—reverses cachexia-induced FASN downregulation, significantly reduces serum FFA levels, and increases triglyceride levels 3—significantly improve the symptoms of weight loss and fat atrophy caused by cachexia	[67]
Curcumin+bis-1,7-(2-hydroxyphenyl)-hepta-1,6-diene-3,5-dione (BDMC-A)		mouse model	Lipid metabolism	1—reduce cholesterol in the colon and intestine 2—enhance fecal cholesterol and bile acid excretion and reverse intestinal lipid accumulation 3—phospholipid content recovery and PLA and PLC activity inhibition in colon and intestine	[68]

**Table 3:** The regulatory effect of Curcumin on tumor arachidonic acid metabolism.

Diesase	Type of Cell Lines or Animal Model	Regulatory Ways	Main Findings	Refs
CRC	HT-29, HCT-116	AA metabolism	1—inhibits cPLA2 phosphorylation, COX-2, and 5-LOX 2—block the release of arachidonic acid metabolism products	[72]
			1—activated AMPK inhibits Akt and COX-2 expression 2—inhibits cell proliferation and triggers apoptosis	[73]
	mouse model	AA metabolism	1—decreased production of prostaglandins (PGE2, PGE2α, PGD2, 6-keto-PGF1α) and thromboxane (TXB2) in liver and colon 2—inhibits the production of LOX pathway products 5 (s)-, 8 (s)-, 12 (s), and 15 (s)-HETEs	[74]
			1—down regulated the levels of phospholipase A2, phospholipase Cγ1 and PGE2 in colon mucosa and tumor tissues 2—inhibit COX and LOX metabolism, both reduce COX-mediated PGE2, PGF2α, PGD2, 6-keto PGF1α, and TXB2 synthesis, and reduce various types of hemes generated by the LOX pathway	[75]
ESCC	KYSE-150, KYSE-450	AA metabolism	1—inhibits cPLA2 phosphorylation, COX-2, and 5-LOX 2—block the release of arachidonic acid metabolism products	[72]
AML	HL-60	AA metabolism	inhibit COX-2 expression and trigger mitochondrial apoptosis	[76]
BC	MCF-7, MCF-7R	AA metabolism	inhibition of Bcl-2 and COX-2 expression inhibits tumor proliferation	[77]
	MCF10A	AA metabolism	1—block ERK1/2 and NF-kB transcriptional activity 2—inhibited TPA-induced upregulation of COX-2 and MMP-9 3—decreased PGE2 synthesis and antagonized tumor metastasis and invasion	[78]
Lung cancer	A549	AA metabolism	inhibit microsomal PGE2 synthase-1 activity and block PGH2 to PGE2 conversion	[79]
			1—inhibits EGR-1, NF-kB, and JNK1/2 signaling 2—blocked IL-1β induced mPGES-1 and COX-2 expression and inhibited PGE2 biosynthesis	[80]
	PC-14	AA metabolism	inhibition of COX-2, EGFR and ERK1/2 activity and expression	[81]
	mouse model	AA metabolism	inhibition of NF-kB and COX-2 activity inhibits tumor growth	[82]
Pancreatic cancer	Panc-1	AA metabolism	inhibition of COX-2, EGFR and ERK1/2 activity and expression	[81]
	mouse model	AA metabolism	decreased the expression of iNOS, COX-2, and 5-LOX in tumors	[83]
CC	HeLa, SiHa, C33A	AA metabolism	inhibition of NF-kB, COX2, and AP-1 expression	[84]
	mouse model	AA metabolism	inhibit VEGF, COX-2, and EGFR, inhibit tumor growth and angiogenesis	[85]
Skin cancer	mouse model	AA metabolism	downregulate NF-kB, COX-2, PGE2, and NO, and inhibit UVB radiation-induced inflammation and carcinogenesis	[86]
HNC	Hep-2, CNE-1, HNE-2, 212LN, SCC38	AA metabolism	1—activated AMPKα and p38MAPK, promoted PGC-1α protein expression and reduced SP1 levels 2—inhibiting the PGE2 receptor EP4 gene expression to inhibit cancer cell proliferation	[87]
OC	AMOS-III	AA metabolism	inhibits the activation of NF-κB and COX-2 induced by NNK	[88]
Melanoma	4046T	AA metabolism	inhibit NF-kB and downstream COX-2 and cyclin D1 expression	[89]
HCC	mouse model	AA metabolism	decreasing COX-2 and VEGF signaling inhibits tumor angiogenesis	[90]

**Table 4:** The regulatory effect of Curcumin on tumor ferroptosis.

Compound	Diesase	Type of Cell Lines or Animal Model	Regulatory Ways	Main Findings	Refs
Curcumin	ALL	Nalm-6	Ferroptosis, Redox metabolism	1—promote ACSL4 expression and reduce SLC7A11 and GPx4 expression 2—induced GSH depletion, lipid peroxidation, ROS generation, and Fe^2+^ accumulation	[99]
Curcumin	BC	MCF-7, MDA-MB-231	Ferroptosis, Redox metabolism , Mitochondrial metabolism	1—up regulation of FTL, FTH1, and TFRC expression 2—downregulated GPx4 and upregulated HO-1 and Nrf2 levels 3—promote ROS overproduction, leading to increased intracellular iron ROS, lipid peroxide, and MDA levels, while GSH levels decreased	[96]
				1—promotes HO-1 hyperactivation leading to increased intracellular levels of Fe^2+^, MDA, LOOH, and ROS 2—decreased FHC, GPx4 levels, and GSH content, resulting in an imbalance of Fe^2+^ levels and loss of MMP	[100]
		MDA-MB-453, MCF-7, mouse model	Ferroptosis, Redox metabolism	1—up regulation of SLC1A5 enhances glutamine uptake 2—promoted MDA elevation and Fe^2+^ accumulation, but GSH content decreased	[101]
		MCF-7, mouse model	Ferroptosis, Mitochondrial metabolism, Redox metabolism	1—promote Ca^2+^ overload to destroy MMP and activate apoptosis 2—H_2_O_2_ generated by the 2-reaction promotes the disintegration of TF structure to release Fe^3+^ to induce ferroptosis	[102]
				1—depletion of GSH triggered ROS generation and promoted OH-1 expression to produce large amounts of Fe^2+^ 2—decreased GPx4 expression and induced cellular lipid peroxidation and ferroptosis	[103]
		4T1	Ferroptosis, Redox metabolism	1—promote ROS generation, disrupt MMP, and induce apoptosis 2—deplete GSH and downregulate GPx4 expression to activate ferroptosis	[104]
Curcumin analog, 4d		MCF-7	Ferroptosis, Redox metabolism	1—promote Fe^2+^ accumulation and increase ROS and MDA levels 2—inhibition of SLC7A11/GPx4 signaling induces ferroptosis	[105]
Curcumin	GC	SGC-7901, MGC-803, mouse model	Ferroptosis, Redox metabolism	up regulation of HO-1 and inhibition of GPx4 expression triggered lipid peroxidation, resulting in increased MDA levels, iron accumulation, ROS generation, and GSH depletion	[106]
		AGS, HGC-27	Ferroptosis, Redox metabolism	1—inhibition of PI3K/Akt/mTOR signaling induced autophagy, and the levels of its markers ATG5, ATG7, Beclin 1, and LC-3B increased 2—promoted the upregulation of intracellular iron, MDA, and ACSL4 levels, and the downregulation of lipid ROS, SLC7A11, GSH, and GPx4 levels	[107]
Curcumin	HCC	HepG2, SMMC-7721, mouse model	Ferroptosis, Redox metabolism	1—decreased GPx4 and SLC7A11 expression and promoted ACSL4 and PTGS2 expression 2—increases intracellular MDA and Fe^2+^ levels, decreases GSH levels, and increases ferroptosis sensitivity	[108]
		PLC, KMCH, Huh 7	Ferroptosis	induced changes in the expression of metal ion homeostasis-related genes (CYP1A1, HMGCS2, HMOX1, LCN2, and MTTP) and activated ferroptosis	[109]
Curcumin	Lung cancer	CD133^+^A549, mouse model	Ferroptosis	decreased GPx4 and FSP1 expression, induced ferroptosis by inhibiting GSH-GPx4 and FSP1-CoQ 10-NADH pathways	[110]
Curcumin+ quercetin		cell and mouse model	Ferroptosis	regulating miR-520a-5p/SLC7A11 signaling inhibits circFOXP1 expression	[111]
Curcumin		A549, H1299, mouse model	Ferroptosis, Redox metabolism	1—increased MDA content, decreased SOD activity, promoted GSH consumption, and increased iron content in tumor tissues 2—upregulated ACSL4 and downregulated the expression of SLC7A11 and GPx4 3—activating autophagy promotes the occurrence of ferroptosis	[112]
Curcumin		16HBE, LK-2, H1650, mouse model	Ferroptosis, Redox metabolism	1—downregulate SOD and GSH levels, upregulate MDA, LDH, and Fe^2+^ levels 2—downregulated SLC7A11 and GPx4 levels, and upregulated ACSL4 and TfR1 levels	[113]
Curcumin analog, HO-3867		H460, A549	Ferroptosis, Redox metabolism	promote iron accumulation and ROS generation, induce ferroptosis by activating p53-DMT1 signaling, and inhibit GPx4	[114]
Curcumin analog, 2c		NCI-H460	Ferroptosis, Redox metabolism	1—inhibiting TrxR activity promotes ROS generation and triggers GSH depletion 2—induced inactivation of GPx4, leading to intracellular lipid peroxidation and MDA accumulation	[115]
Curcumin	CRC	HCT116	Ferroptosis	induction of ferroptosis	[116]
		SW-480	Ferroptosis, Redox metabolism	decreased Myc, IL-1β, and EZH2 mRNA expression, and promoted SLC1A5 and CAV1 expression	[95]
				inhibition of JNK induced downregulation of GPx4 and FTH1, upregulation of ACSL4 led to intracellular ROS and iron accumulation, while the level of lipid peroxidation increased	[117]
		HCT-8	Ferroptosis, Redox metabolism	1—downregulate GSH, SLC7A11 and GPx4 levels through PI3K/Akt/mTOR signaling 2—promote the increase in ROS, MDA, and iron content to trigger ferroptosis	[5]
		SW620, LoVo, mouse model	Ferroptosis, Redox metabolism	1—activating p53 and inhibiting SLC7A11/GSH/GPx4 axis to induce ferroptosis 2—upregulation of LDH release, ROS, lipid peroxide, Fe^2+^ accumulation, and MDA level, and synchronously downregulated GSH and GPx4 activities	[118]
Curcumin+Andrographis paniculata		SW480, HCT116	Ferroptosis	inhibition of GPx4 and FSP-1 expression activates ferroptosis	[119]
Curcumin+Metformin		CT 26, HCT 116	Ferroptosis, Redox metabolism	reduce xCT-GPx4 axis activity and downregulate DMT1 expression to promote cellular lipid peroxidation	[120]
Curcumin analog, ALZ003	GBM	U87MG, Pt#3	Ferroptosis, Redox metabolism	induction of FBXL2-mediated AR ubiquitination down regulated GPx4 expression, resulting in lipid peroxidation and ROS accumulation	[121]
Curcumin	FTC	FTC-133, FTC-238	Ferroptosis, Redox metabolism	induced HO-1 hyperactivation and decreased GPx4 expression, promoted significantly increased intracellular iron, MDA, and lipid ROS content, and decreased GSH content	[122]
Curcumin	OS	MNNG/HOS, MG-63, mouse model	Ferroptosis, Redox metabolism	decreased the expression levels of Nrf2, SLC7A11, HO-1, and GPx4, resulting in increased ROS and MDA levels and decreased GSH levels	[123]
Curcumin analog, EF24		U2os, Saos-2	Ferroptosis, Redox metabolism	upregulate HMOX1 and inhibit GPx4 expression, promote intracellular MDA, ROS and Fe^2+^ levels	[124]
Curcumin	ccRCC	786-O-DR	Ferroptosis, Redox metabolism	reduce the intracellular iron concentration and reduce the expression of NCOA4, FTH1 and p53 by up regulating ADAMTS18 gene	[15]
Curcumin analog, MitoCur-1	Melanoma	A375, SKMEL28	Ferroptosis, Redox metabolism	1—inhibition of SLC7A11 blocks cystine uptake, resulting in reduced GSH synthesis 2—inhibition of the deubiquitinating enzyme USP14 reduces GPx4 protein expression and synergistically promotes abnormal accumulation of lipid ROS and Fe ^2+^	[125]
Curcumin analog, NL01	OC	Anglne, HO 8910 PM	Ferroptosis, Energy metabolism	1—reduced GPx4 expression induces ferroptosis 2—decreased HCAR1/MCT1 expression blocks lactate transport capacity and activates AMPK to inhibit SREBP signaling	[126]

**Table 5:** The regulatory effect of Curcumin on tumor amino acid metabolism.

Compound	Diesase	Type of Cell Lines or Animal Model	Regulatory Ways	Main Findings	Refs
Curcumin analog, WZ35	GC	BGC-823, SGC-7901	Amino acid metabolism, Mitochondrial metabolism	1—downregulates SLC7A11 and SLC1A5 expression, reduces glutamate and cysteine uptake, and multidimensional regulates GSH metabolism 2—activate ROS-YAP-AXL-ALKBH5-GLS2 signal to induce and maintain GSH depletion phenotype, promote GSH reserve depletion and GSH decomposition acceleration by reducing GSH precursor supply and GSSG regeneration, reduce the levels of synergistic branched chain amino acids and other amino acids, and realize the continuous consumption of metabolic substrates	[14]
Curcumin	BC	mouse model	Amino acid metabolism, nucleotide metabolism	1—Nodal metabolites such as chitobiose, D-glucosamine-6-phosphate, L-fucose, and N-acetyl-β-mannosamine showed specific changes 2—in the pathway of amino acid biosynthesis, the levels of amino acids such as DL-isoleucine, DL-tyrosine, and homocysteine fluctuated significantly	[130]
		MDA-MB-453, MCF-7, mouse model	Amino acid metabolism, Ferroptosis	promote SLC1A5 dependent glutamine uptake enhancement, synergistically inhibit FTH and GPx4, and activate ACSL4 and NOX1, forming a multi-target ferroptosis induction network	[101]
Curcumin	CRC	organoid	Amino acid metabolism	regulates the biosynthesis of phenylalanine, tyrosine, and tryptophan, the metabolism of nicotinic acid and nicotinamide, and the metabolism of purine	[131]
		SW 620/Ad 300	Amino acid metabolism, Mitochondrial metabolism	inhibiting ODC expression impairs polyamine synthesis and D-Gln metabolism, reduces glutamate, GSH, and ATP levels to inhibit P-gp efflux activity, promotes intracellular drug accumulation, and induces apoptosis	[4]
		CD44^+^HT29	Amino acid metabolism	reduce glutamine levels and induce apoptosis	[132]
		HT-29	Amino acid metabolism	inducing miR-137 inhibits GLS activity and disrupts glutamine metabolic homeostasis	[133]
		Caco-2	Amino acid metabolism, Redox metabolism, Lipid metabolism	1—upregulate NAD/NADP metabolism related proteins (such as AKR1B10, AKR1C1, AKR1C3, BLVRB, CBR1, CBR3, HSD17B11, ME1, PTGR1) and enhance redox metabolism 2—regulates the expression of proteins related to RNA metabolism and lipid metabolism, such as ACOX1, ACSL1, HMGCS1, and PLIN3 3—upregulate the expression of copper toxicity stress-related proteins MRPS14, GCLM, IMP4, and FAU to mediate the imbalance of copper homeostasis	[134]
Curcumin+Ursolic acid	PC	mouse model	Amino acid metabolism	targeting ASCT2 reduces glutamine uptake	[135]
Curcumin	ACC	H295R, SW13, MUC-1	Amino acid metabolism, Ferroptosis	upregulation of SLC1A5 and gls1 expression forces tumor cells to rely on glutamine metabolism, resulting in ROS overproduction, triggering ferroptosis	[46]
Curcumin	Myeloid leukemia	Kasumi-1, KG-1, HL60	Amino acid metabolism	inhibit BCAT1 expression, synergistically inhibit mTOR signaling, and reduce α-KG levels	[136]
Curcumin+Gemcitabine	CCA	KKU-213B, mouse model	Amino acid metabolism, Mitochondrial metabolism	inhibition of LAT2 reduces Gln and synergistically downregulates GLS and GS expression in combination with gemcitabine, resulting in reduced Glu production and blocking its participation in key metabolism, such as nucleotide synthesis and TCA cycle	[137]

**Table 6:** The regulatory effect of Curcumin on tumor polyamine metabolism.

Diesase	Type of Cell Lines or Animal Model	Regulatory Ways	Main Findings	Refs
Skin cancer	mouse model	Polyamine metabolism	inhibition of epidermal ODC activity and skin tumorigenesis	[92]
			1—inhibited TPA-induced upregulation of ODC mRNA levels 2—accelerate ODC mRNA breakdown to reduce activity	[148]
			1—inhibits TPA-induced PKC translocation, MAPK signaling (JNK, ERK, and p38) activation, and downstream transcription factor (c-jun and c-fos) expression 2—inhibit COX-2 and ODC activities, reduce oxidative damage, 3- inhibit skin inflammation and proliferation, and induce apoptosis	[149]
			eliminate TPA-induced increase in ODC activity, inhibit dermal inflammatory cell infiltration, and tumor progression	[150]
			inhibiting the expression of ODC and metallothionein blocks the tumor-promoting effect of UVA-TPA	[151]
BC	MCF-7, MDA-231	Polyamine metabolism	inhibit ODC activity and cell proliferation	[152]
			inhibit NF-kB signaling and trigger ROS-dependent cell cycle arrest and apoptosis	[153]
APL	HL-60	Polyamine metabolism, Mitochondrial metabolism	1—inhibit ODC activity and expression 2—activated ROS-dependent mitochondrial apoptosis pathway	[140]
CRC	SW 620/Ad 300	Polyamine metabolism	1—inhibit ODC expression, block polyamine synthesis and D-Gln metabolism, and reduce intracellular glutamate, GSH, and ATP levels 2—impairs antioxidant capacity and exhibits pro-apoptotic effects	[4]
	mouse model	Polyamine metabolism, AA metabolism	1—reduce the activity of ODC, TPK, and arachidonic acid metabolism products in the liver and colon mucosa 2—inhibition of ACF formation	[74]
OC	mouse model	Polyamine metabolism	reduce ODC activity and polyamine levels in the oral mucosa	[154]
RCC	mouse model	Polyamine metabolism	alleviate oxidative stress and inhibit the abnormal increase in ODC activity	[155]

## Data Availability

No new data were created or analyzed in this study. Data sharing is not applicable to this article.

## Abbreviations

HIF-1α	hypoxia-inducible factor-1α
GPI	glucose-6-phosphate isomerase
MDH	malate dehydrogenase
ENO1	enolase 1
PDK1	pyruvate dehydrogenase kinase 1
TPI	triose phosphate isomerase
PGK1	phosphoglycerate kinase 1
GAPDH	glyceraldehyde-3-phosphate dehydrogenase
ALDOA	aldolase A
PDH	pyruvate dehydrogenase
MCT	monocarboxylate transporter
G6PD	glucose-6-phosphate dehydrogenase
GLUT	glucose transporter
HK	hexokinase
LDHA	lactate dehydrogenase A
PKM2	pyruvate kinase M2
PGD	phosphogluconate dehydrogenase
PHGDH	phosphoglycerate dehydrogenase
GOT	glutamic-oxaloacetic transaminase
GPX1	glutathione peroxidase 1
SOD2	superoxide dismutase 2
OCR	oxygen consumption rate
GPX4	glutathione peroxidase 4
MMP	matrix metalloproteinase
LDH	lactate dehydrogenase
MDA	malondialdehyde
ROS	reactive oxygen species
FTH	ferritin heavy chain
NOX1	NADPH oxidase 1
PTGS2	prostaglandin-endoperoxide synthase 2
GSS	glutathione synthetase
SLC7A11	solute carrier family 7 member 11
HMGCR	3-hydroxy-3-methylglutaryl-CoA reductase
SREBP	sterol regulatory element-binding protein
BCAT1	branched-chain amino acid transaminase 1
CML	chronic myelogenous leukemia
ACC	adrenocortical Carcinoma
HNSCC	squamous cell carcinoma of head and neck
MM	multiple myeloma
ALL	acute lymphoblastic leukemia
FTC	follicular thyroid cancer
ccRCC	clear cell renal cell carcinoma
CACS	cancer anorexia-cachexia syndrome
PBMC	peripheral blood mononuclear cell
TAC	total antioxidant capacity
DMC	demethylated curcumin
ATG	autophagy-related protein
LDLR	low-density lipoprotein receptor
FASN	fatty acid synthase
GPAT1	glycerol-3-phosphate acyltransferase 1
GPAM	glycerol-3-phosphate acyltransferase
SCD	stearoyl-CoA desaturase
ACC	acetyl-CoA carboxylase
ACLY	ATP citrate lyase
COX	cyclooxygenase
LOX	lipoxygenase
PTDSS1	phosphatidylserine synthase 1
SLC13A5	solute carrier family 13 member 5
FADS1/2	fatty acid desaturase 1/2
NPC1L1	niemann-pick c1-like 1
MVD	mevalonate diphosphate decarboxylase
SQLE	squalene epoxidase
FDPS	farnesyl diphosphate synthase
FABP1	fatty acid binding protein 1
PGD2	prostaglandin D2
GLS	glutaminase
GS	glutamine synthetase
LAT2	L-type amino acid transporter 2
ODC	ornithine decarboxylase
TPK1	thiamin pyrophosphokinase 1
COX-2	cyclooxygenase-2
SCFA	short-chain fatty acid
BCFA	branched-chain fatty acid
FFA	free fatty acid
PPP	pentose phosphate pathway
TCA	tricarboxylic acid cycle
OXPHOS	oxidative phosphorylation
GSH	glutathione
HO-1	heme oxygenase 1
SOD	superoxide dismutase
BC	breast cancer
GC	gastric cancer
CRC	colorectal cancer
GBM	glioblastoma
HCC	hepatocellular carcinoma
NBL	neuroblastoma
RCC	renal cell carcinoma
CC	cervical cancer
DL	dalton’s Lymphoma
ESCC	esophageal squamous carcinoma
PTC	papillary thyroid carcinoma
